# How atoms of polycrystalline Nb_20.6_Mo_21.7_Ta_15.6_W_21.1_V_21.0_ refractory high-entropy alloys rearrange during the melting process

**DOI:** 10.1038/s41598-022-09203-y

**Published:** 2022-03-25

**Authors:** Shin-Pon Ju, Chen-Chun Li, Huai-Ting Shih

**Affiliations:** 1 Department of Mechanical and Electro-Mechanical Engineering, National Sun Yat-Sen University, Kaohsiung, 804 Taiwan; 2 Department of Medicinal and Applied Chemistry, Kaohsiung Medical University, Kaohsiung, 807 Taiwan

**Keywords:** Engineering, Materials science, Nanoscience and technology

## Abstract

The melting mechanism of single crystal and polycrystalline Nb_20.6_Mo_21.7_Ta_15.6_W_21.1_V_21.0_ refractory high entropy alloys (RHEAs) were investigated by the molecular dynamics (MD) simulation using the second-nearest neighbor modified embedded-atom method (2NN MEAM) potential. For the single crystal RHEA, the density profile displays an abrupt drop from 11.25 to 11.00 g/cm^3^ at temperatures from 2910 to 2940 K, indicating all atoms begin significant local structural rearrangement. For polycrystalline RHEAs, a two-stage melting process is found. In the first melting stage, the melting of the grain boundary (GB) regions firstly occurs at the pre-melting temperature, which is relatively lower than the corresponding system-melting point. At the pre-melting temperature, most GB atoms have enough kinetic energies to leave their equilibrium positions, and then gradually induce the rearrangement of grain atoms close to GB. In the second melting stage at the melting point, most grain atoms have enough kinetic energies to rearrange, resulting in the chemical short-ranged order changes of all pairs.

## Introduction

Materials used in the extreme working environments such as high temperatures or pressures are in an urgent need for industrial application. For example, to improve the efficiency of gas turbine engines in the aerospace industry, increasing engine operating temperature is one of the most effective ways^1^. However, the most commonly used high-temperature structural material, nickel-based superalloy, has its own melting point of about 1300 °C, which limits the maximum operating temperature^2,3^. Thus, it is very important that the material has a high enough melting point^4^. High entropy alloys (HEAs), also known as multi-principal-element alloys (MPEAs), are composed of major element types more than four^5^. Within HEAs, all compositional elements are arranged in the most uniformly distribution, leading to excellent material properties including high hardness^6^, high strength and ductility combination^7,8^, good fatigue resistance^9^, high temperature microstructure and mechanical stability^10^, outstanding electromagnetic properties^11^, excellent wear resistance^12^, corrosion resistance^13^, and oxidation resistance^14^.

Among all HEAs, the refractory high entropy alloys (RHEAs) generally have one or more compositional refractory elements such as W, Mo, Ta, Nb, Zr, and Re^15^. For HEAs without refractory elements, the poor phase stability and low plasticity at high and medium temperatures are two bottlenecks to restrict their applications at high temperatures. Accordingly, RHEAs display excellent high temperature resistance, high melting point (> 2000 °C), and higher high temperature strength, which has wide potential for applications in high temperature equipment. For examples, in 2010, the first RHEA, NbMoTaW RHEA, was fabricated by Senkov^16^*.* The yield strength of NbMoTaW RHEA at 1600 °C is 405 MPa, and the working temperature limit of 1600 °C is much higher than that of nickel-based high temperature alloys about 1300 °C. In Xia’s study^17^, the thermal stability of MoNbTaVW RHEA thin film was studied, and the experimental results show the body-centered cubic solid solution phase of MoNbTaVW RHEA is still very stable up to 1800 K. In Zhang’s experimental study^18^, the plastic deformation mechanism of MoNbTaVW RHEA under high pressure was observed. It was found the active dislocation growth is mainly responsible for the high strength in the MoNbTaVW RHEA. In Yang’s study^19^, they found an effective way to improve the oxidation resistance of MoNbTaVW RHEA by using Si/Al pack cementation coatings, which also improves the mechanical properties of MoNbTaVW RHEA at high temperatures. In Nie’s study^1^, the HfMoScTaZr RHEAs were prepared by vacuum arc melting equipment. By adding Sc element, the density of the alloy becomes lower, and the strength and plasticity of HfMoScTaZr RHEAs were significantly improved. The yield strengths of HfMoScTaZr RHEAs at room temperature, 800 °C, 1000 °C and 1200 °C are 1778, 1118, 963 and 498 MPa, respectively. At 1200 °C, the yield strength of HfMoScTaZr RHEA is about 4.3 and 6 times higher than those of the traditional classic superalloys, Inconel 718^20^ and CMSX-4^21^. Besides the compositional element types and their related fractions, the material properties of HEAs or alloys are significantly affected by the extent of crystallinity. For examples, In Lin's study^22^, the melt-ball milling-hot pressing process was adopted to fabricate the Cu_3−x_Ni_x_SbSe_4_ (x = 0–0.03) alloys with different average grain sizes. The influences of average grain size on the microstructure and thermoelectric properties of Cu_3−x_Ni_x_SbSe_4_ were observed. Because of the grain refinement and Se defect increase, the lattice thermal conductivity decreases from 3.3 W m^−1^ K^−1^ to 2.4 W m^−1^ K^−1^ at room temperature when the fraction Ni fraction decreases from x = 0.03 to 0. In Sun’s study^23^, they experimental results indicate when the grain size of CoCrFeMnNi HEA decreases from 105 μm to 650 nm at 293 K, the yield strength increased from 225 to 798 MPa with an increase of 254.7%. At the same time, the ultimate tensile strength increases from 798 to 887 MPa with an increase of 11.2%. Bhandari et al*.* adopted the density functional theory (DFT) method was to compute the structural and mechanical properties of AlCrMoTiV^3^. According to the DFT prediction, the Al_30_Cr_10_Mo_5_Ti_20_V_35_ RHEA has the optimal element fractions to possess a lower density of 5.16 g/cm^3^ and a higher hardness of 5.56 GPa.

For new RHEA development, it is essential to understand the thermal behavior of single crystal and polycrystalline RHEAs from the atomic scale. Using the empirical approach, it is relatively difficult to directly observe the atomic arrangement and diffusion at high temperatures. Consequently, the molecular simulation method has played an important role to investigate the atomic behaviors at high temperatures or during the melting process. For examples, in Rahman’s MD simulation results^24^, they found the steady-state creeping rate of nanocrystalline Cu_0.5_Ni_0.5_ alloy speeds up dramatically under the elevated stress and temperature as well as the decreasing grain size. The lattice and grain boundary diffusion play a critical factor of creeping deformation mechanism of the nanocrystalline Cu_0.5_Ni_0.5_ alloy. Giang et al*.* studied the melting stages of 2D confined germanene in both perfect crystalline and polycrystalline states by MD simulation^25^. The temperatures from solid to liquid phase transition are about 1670 K and 1540 K for the crystalline and polycrystalline models, respectively. In Noori’s study^26^, the MD simulation was utilized to realize the effect of grain size on the melting temperature of Al nanocrystals. Their results show that the melting temperature becomes lower when the grain size is smaller. The pre-melting and melting at grain boundary regions does not take place instantly, and the melting of the polycrystalline Al occurs within a certain temperature range, rather than at a specific temperature.

The systematic investigation on how atoms rearrange for a single crystal and polycrystalline RHEAs during the heating process is still lacking. In order to explore the melting mechanism, the single crystal Nb_20.6_Mo_21.7_Ta_15.6_W_21.1_V_21.0_ RHEA and polycrystalline Nb_20.6_Mo_21.7_Ta_15.6_W_21.1_V_21.0_ RHEAs with the average grain sizes from 5.2 to 25.3 nm were used for the MD heating simulation from 300 to 3600 K. The second-nearest neighbor modified embedded-atom method (2NN MEAM) potential was used to model the interaction among Nb, Mo, Ta, W, and V elements. The system enthalpy and square displacement at different temperatures of the heating process were used to determine the melting points of Nb_20.6_Mo_21.7_Ta_15.6_W_21.1_V_21.0_ RHEAs. The dynamical behaviors of different elements at grain boundaries and within grains were also investigated, and variations of affinity between any two element type pairs were investigated by the chemical short-ranged order during the heating process.

## Results and discussion

**Figure 1:**
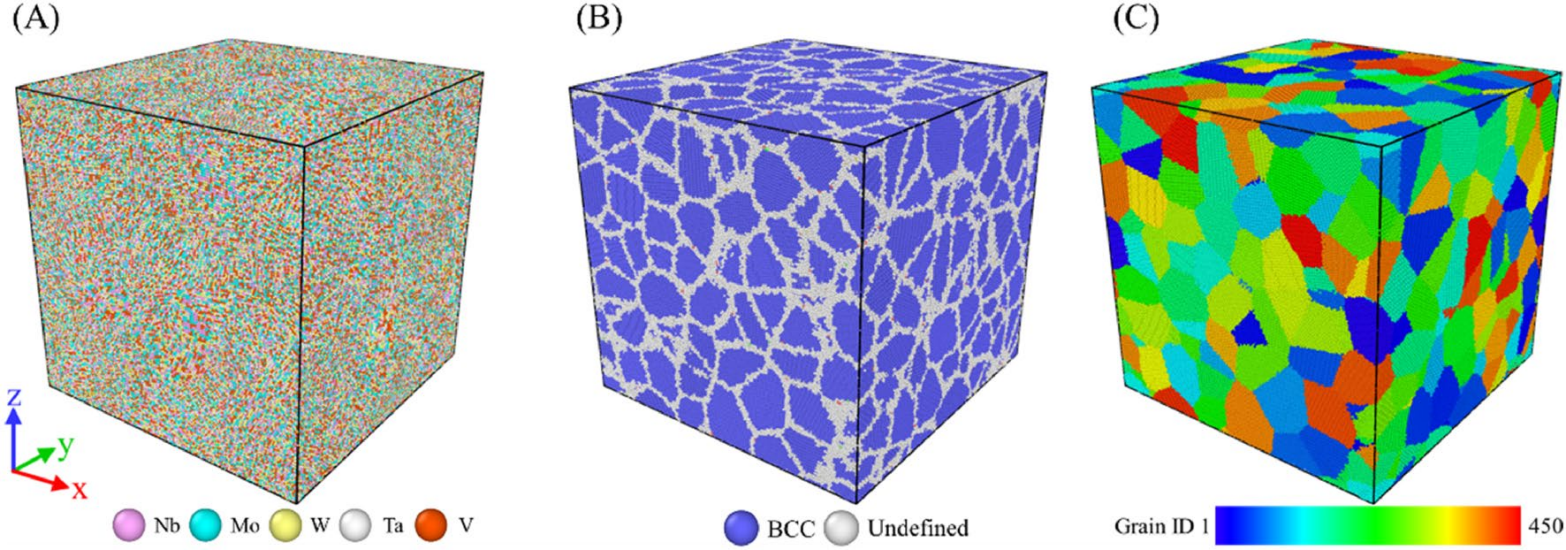
The polycrystalline Nb_20.6_Mo_21.7_Ta_15.6_W_21.1_V_21.0_ RHEA model with the average grain size about 5.2 nm for the heating process. The system size is about 40.7, 40.7, and 40.7 nm in the x-, y-, and z-dimensions, respectively. Atoms are colored according to (**a**) the element type, (**b**) grain and grain boundary atoms identified by the common neighbor analysis (CNA), and (**c**) the grain identity number. The current study considers the RHEAs with the average sizes of 25.3, 20.1, 15.6, 10.0, and 5.2 nm.

**Figure 2:**
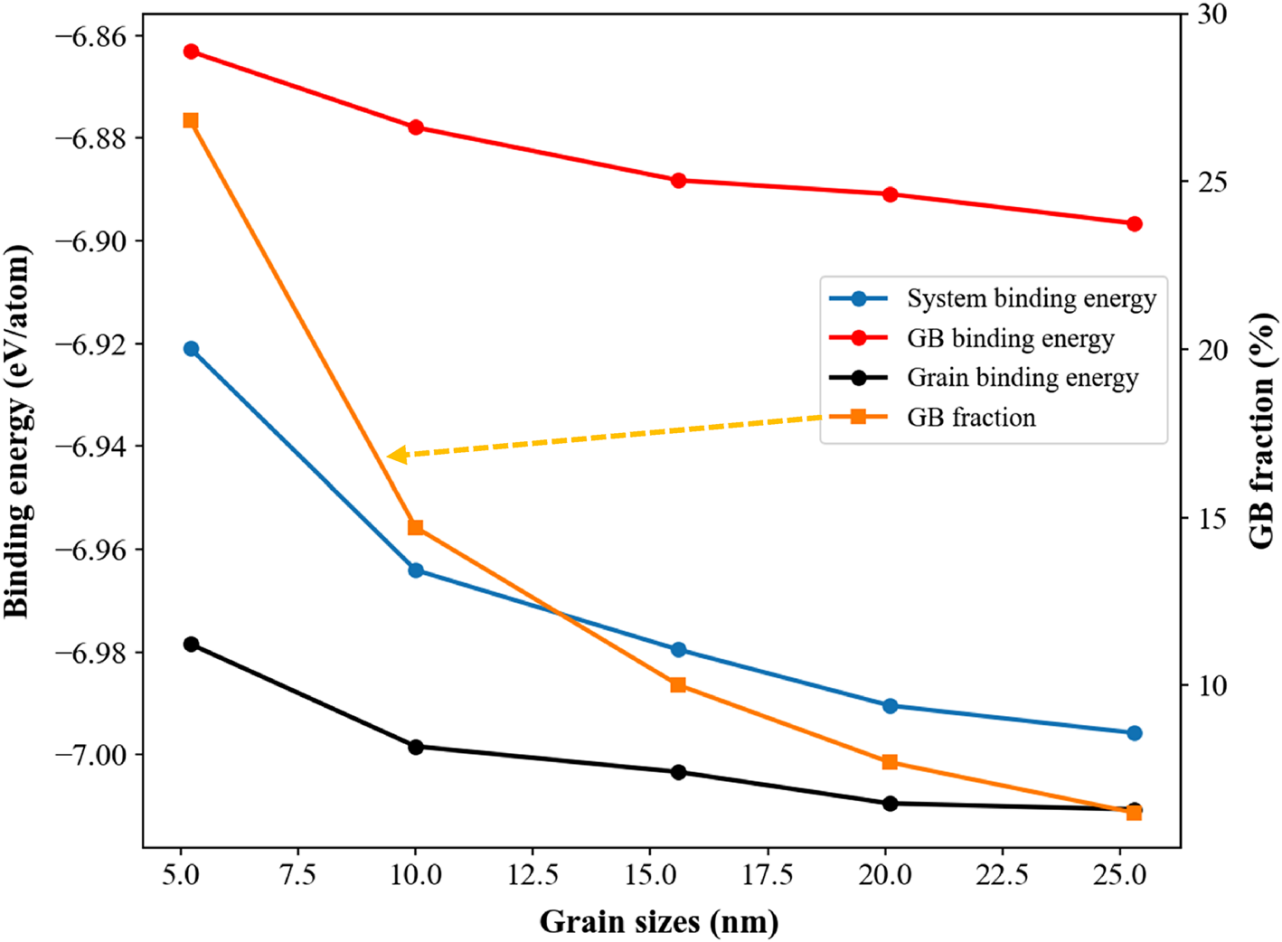
Profiles of GB fraction and the binding energies of grain, GB, and system for Nb_20.6_Mo_21.7_Ta_15.6_W_21.1_V_21.0_ RHEAs with the average grain size from 5.2 to 25.3 nm.

Figure 2 shows the variations of GB atom fractions and atomic binding energies of GB, grain, and system for Nb_20.6_Mo_21.7_Ta_15.6_W_21.1_V_21.0_ RHEAs with the average grain size from 5.2 to 25.3 nm. It can be seen from Fig. 2 that the GB atom fraction decreases parabolically from 26.8 to 6.2% when the average grain size increases from 5.2 to 25.3 nm. In Fig. 1, according to the CNA results, atoms within the GB and grain are arranged in the undefined type and the BCC type, respectively. When the average grain size becomes smaller, the surface area to volume ratio of grains becomes higher. This result can also be seen for a nanoparticle, which the surface area to volume ratio becomes higher for a smaller nanoparticle. Accordingly, the fraction of GB atoms surrounding grains significantly increases for a smaller grain. Atoms at GB/grain interface possess higher local stresses and higher binding energy, so the atomic binding energy of GB, grain, and system decrease parabolically when the grain size increases from 5.2 to 25.3 nm as illustrated in Fig. 2. Because melting behaviors of Nb_20.6_Mo_21.7_Ta_15.6_W_21.1_V_21.0_ RHEAs with the average grain sizes from 5.2 to 25.3 nm are basically similar, only simulation results for the grain sizes of 25.3 and 5.2 nm were discussed. For comparison, the melting process of single crystal Nb_20.6_Mo_21.7_Ta_15.6_W_21.1_V_21.0_ RHEA was also investigated.

The Warren–Cowley chemical short-range-order analysis^27^ for Nb_20.6_Mo_21.7_Ta_15.6_W_21.1_V_21.0_ RHEAs was used to quantify the attraction and repulsion between different elemental pairs and monitor the local structural rearrangement during the heating process. The chemical affinities of a referenced atom with its first neighbor atoms are evaluated by the Warren–Cowley short-range-order parameter, which can quantify the local short-ranged order. The definition of Warren–Cowley short-range-order parameter is shown in the following equation:

1 $${\alpha }_{ij}=1 - \frac{{N}_{ij}}{{c}_{j}{N}_{i}},$$

where ${\alpha }_{ij}$ is the short-ranged order parameter of the i-type referenced atom relative to j-type atom, *N*_*ij*_ is the partial coordination number (CN) for the i-type referenced atom relative to j-type atom obtained from the predicted structure, and *c*_*j*_ and *N*_*i*_ are the fractions of j-type atom within the alloy and the average CN of i-type atoms, respectively. The value of *c*_*j*_ by *N*_*i*_ is an ideal partial CN for the referenced i-type atom relative to the first neighbor j-type atom, and this value completely depends on the respective atomic composition fraction of Nb_20.6_Mo_21.7_Ta_15.6_W_21.1_V_21.0_ RHEA. The second term of Eq. (1) is the ratio of actual CN (N_ij_) to ideal CN (${c}_{j}{N}_{i}$) for the i-type reference atom to its first neighbor j-type atom. If this ratio is larger than 1, it means the affinity of j-type atom to i-type atom in the predicted structure is higher than that in the ideal structure. On the other hand, if this ratio is lower than 1, the affinity of j-type atom to i-type atom in the predicted structure is lower than that in the ideal structure. If the ratio is close to 1, it infers the affinity of j-type atom to i-type atom in the predicted structure is close to that in the ideal structure. Consequently, the positive and negative values of short-ranged order indicate the lower and higher affinity of the element type pair, compared with their ideal affinity. In previous related MD studies for HEA and BMG^28–30^, the Warren–Cowley parameters were used to quantify short-ranged order, indicating the affinity of the element type pair, compared with that of the corresponding element type pair in the ideal uniform distribution model.

**Figure 3:**
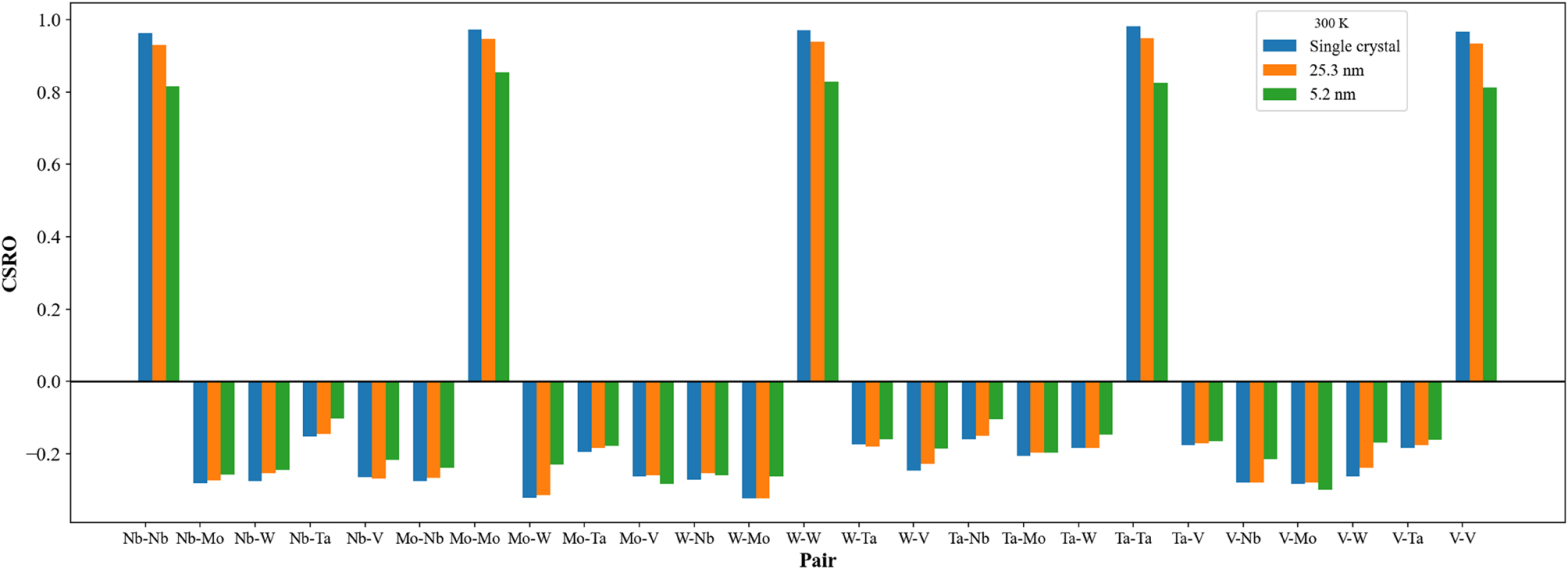
The Nb_20.6_Mo_21.7_Ta_15.6_W_21.1_V_21.0_ RHEA CSRO distributions for (**a**) single crystal, (**b**) 25.3 nm, and (**c**) 5.2 nm at 300 K.

Figure 3 shows the short-ranged order distributions of all element type pairs for single crystal, grain sizes of 5.2 and 25.3 nm at 300 K. All atoms within these three structures are arranged according to the MaxEnt theory, and the distance of the minimum between the first and second peaks of the radial distribution function (RDF) was used to determine short-ranged order values. In Fig. 3, short-ranged order values of pairs with the same element type are larger than 0.8, indicating all element types of Nb_20.6_Mo_21.7_Ta_15.6_W_21.1_V_21.0_ RHEAs are arranged in the most uniform distribution. For pairs with different element types, all short-ranged order values are smaller than − 0.1, indicating the affinity of all pairs with different element types is much higher than those with the same element types.

**Figure 4:**
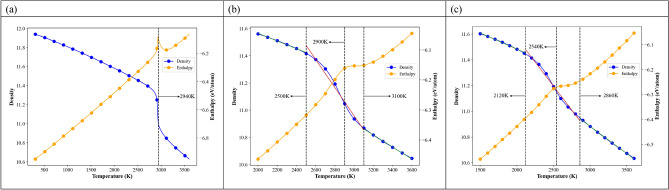
Density and enthalpy profiles of Nb_20.6_Mo_21.7_Ta_15.6_W_21.1_V_21.0_ RHEAs during the heating process for (**a**) single crystal, (**b**) 25.3 nm, and (**c**) 5.2 nm. The melting points of single crystal, 25.3 nm, and 5.2 nm are 2940, 2900, and 2540 K as indicated by the dashed lines. The temperatures at discontinuities of density profiles of two polycrystalline RHEAs are also indicated by the dashed lines.

The first neighbor distance of a reference atom is required to calculate the Warren–Cowley short-ranged order parameter during the MD heating process, so the RDFs at different temperatures were calculated first for obtaining the distances at the minimum between the first and second peaks of RDFs. These distances are critical values to obtain the first neighbor atoms of a reference atom at different temperatures. Then the Warren–Cowley short-ranged order parameters of all element pairs at different temperatures were determined for reflecting the local short-ranged order changes. Figure 4a–c show the variations of density and enthalpy for single crystal, 25.3 nm, and 5.2 nm during the heating process. Because the enthalpies (densities) increase (decrease) linearly with the increasing temperature from 300 K to specific temperatures for all cases, the lowest temperatures of the horizontal axes in Fig. 4b,c start from higher values for clearly showing the variations of density and enthalpy near the melting points. For the single crystal, the enthalpy increases linearly with the increasing temperature from 300 to 2910 K and then shows an abrupt increase from 2910 to 2940 K, within which the local structure of single crystal Nb_20.6_Mo_21.7_Ta_15.6_W_21.1_V_21.0_ RHEA undergoes significantly rearrangement. Consequently, the melting temperature of single crystal Nb_20.6_Mo_21.7_Ta_15.6_W_21.1_V_21.0_ RHEA is 2940 K, which is very close to the predicted value about 2946 K using the rule of mixture^31^. From 2940 to 3110 K, the enthalpy decreases parabolically, and then increases linearly with the increasing temperature when the temperature continuously increases from 3110 K. In Fig. 3 for the single crystal, the short-ranged order values of pairs with the same element type indicate the lowest affinity for atoms to the same element types before the melting point. When the system temperature is higher than the melting point of 2940 K, the atoms have enough kinetic energies to leave their equilibrium positions, and the elements possessing higher binding energies begin to aggregate together, resulting in the decrease of enthalpy from 2940 to 3110 K. For the density profile, it decreases linearly with the increasing temperature from 300 to 2910 K, and then, from 2910 to 2940 K, the density displays an abrupt drop from 11.25 to 11.00 g/cm^3^, indicating the system undergoes the significant local structural rearrangement. In Fig. 4b,c, enthalpy profiles of 25.3 nm and 5.2 nm increase linearly with the increasing temperature from 2100 to 2900 K and from 1500 to 2540 K, respectively. When temperatures continuously increase from 2900 to 3100 K for 25.3 nm and from 2540 to 2700 K for 5.2 nm, enthalpies are almost unchanged. Within these temperature ranges, local structures undergo significantly rearrange. For the density profiles, the discontinuities at 2500 and 3100 K for 25.3 nm and the discontinuities at 2120 and 2860 K for 5.2 nm infer the local structural rearrangement smoothly proceeds during a wider temperature ranges, compared with the density drop within a narrow temperature range (2910–2940 K) for the single crystal. The temperatures of 2900 K and 2540 K are regarded as the melting points of 25.3 nm and 5.2 nm.

**Figure 5:**
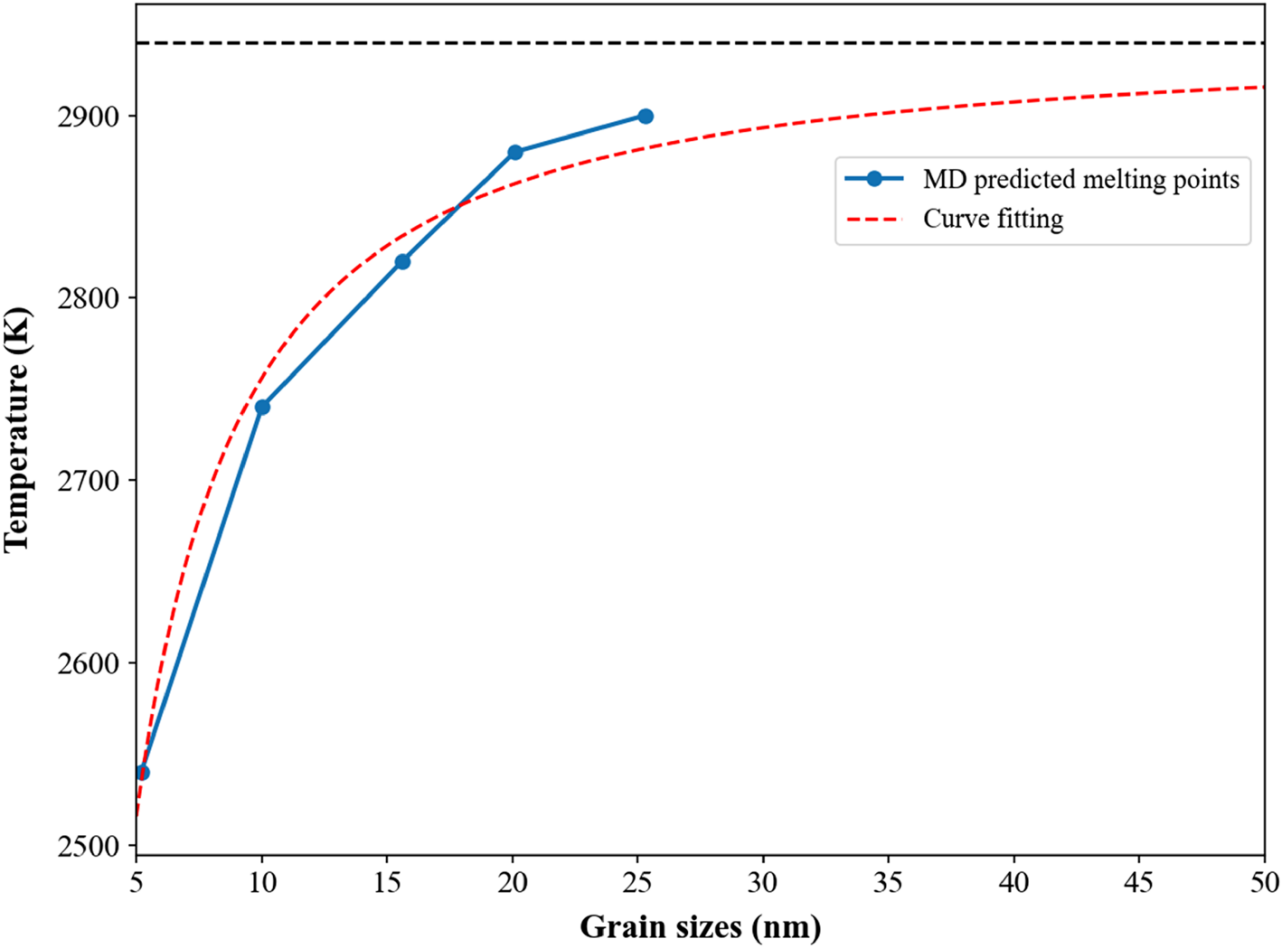
Profile of MD predicted melting points for polycrystalline Nb_20.6_Mo_21.7_Ta_15.6_W_21.1_V_21.0_ RHEAs with the average grain sizes from 5.2 to 25.3 nm. The curve fitting profile using Eq. (2) is also provided. The horizontal dashed line stands for the melting point (2940 K) of the single crystal Nb_20.6_Mo_21.7_Ta_15.6_W_21.1_V_21.0_ RHEA.

Figure 5 shows the profile of the melting point for polycrystalline Nb_20.6_Mo_21.7_Ta_15.6_W_21.1_V_21.0_ RHEAs with the average grain sizes from 5.2 to 25.3 nm. The melting points were temperatures at which the slope of the enthalpy profile begins to change. The horizontal dashed line at 2940 K stands for the melting point of the single crystal Nb_20.6_Mo_21.7_Ta_15.6_W_21.1_V_21.0_ RHEA. It is obvious that the melting point and the grain size display the relationship of logarithmic growth. The increase in the melting point by increasing the grain size is more significant for smaller grains. Consequently, an empirical equation shown below can be used to evaluate the melting point of polycrystalline Nb_20.6_Mo_21.7_Ta_15.6_W_21.1_V_21.0_ RHEAs with much larger grain sizes:

2 $${T}_{m}\left(d\right)={T}_{0}\mathrm{exp}\left(a/{d}^{n}\right),$$

where T_m_(d), T_0_, and d are the melting point of the case with the average grain size of d, the melting point of single crystal, and the average grain size, respectively. Two parameters, a and n (the power of d) are determined by the parametrization process using the available data from the MD simulation. The fitted values of a and n are − 1.2 and 1.27, and the profile of the curve fitting is also shown in Fig. 5, demonstrating the melting points of Nb_20.6_Mo_21.7_Ta_15.6_W_21.1_V_21.0_ RHEAs with much larger grain sizes gradually approach the value of the single crystal.

**Figure 6:**
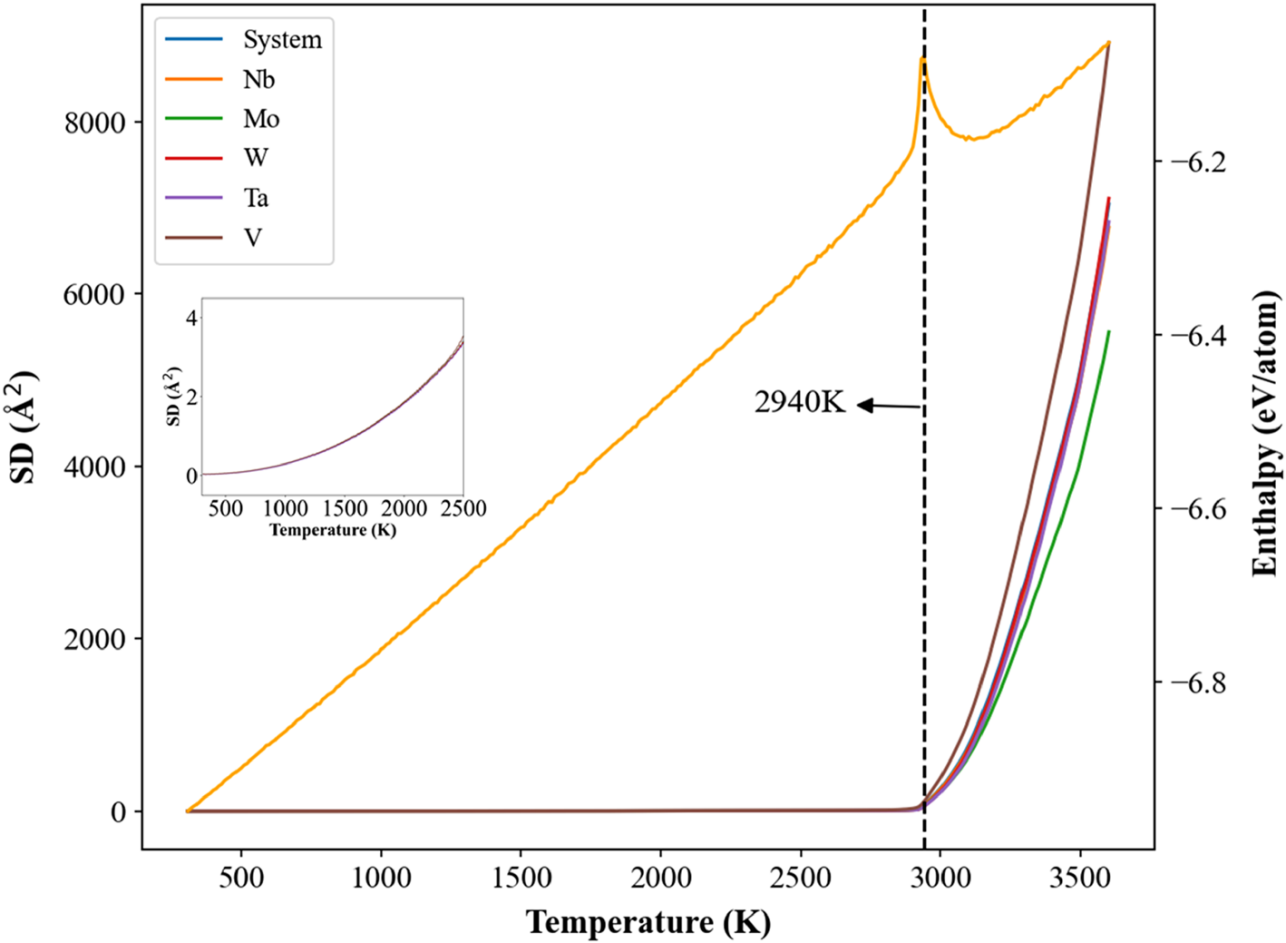
The square displacement (SD) and enthalpy profiles of system, Nb, Mo, W, Ta, and V during the heating process for the single crystal Nb_20.6_Mo_21.7_Ta_15.6_W_21.1_V_21.0_ RHEA. The insert shows SD profiles at temperatures lower than the melting point of 2940 K.

The square displacement (SD) profile during the temperature elevation was used to observe the melting behaviors of single crystal and cases with the grain sizes of 25.3 and 5.2 nm for Nb_20.6_Mo_21.7_Ta_15.6_W_21.1_V_21.0_ RHEAs. The definition of SD at time t is shown in Eq. (3):

3 $$\mathrm{SD}\left(\mathrm{t}\right)=\frac{\sum_{i}^{N}{\left[{r}_{i}\left(t\right)-{r}_{i}\left(0\right)\right]}^{2}}{N},$$

where *r*_*i*_ (0) is the position of the *i-*th atom at time 0, *r*_*i*_ (*t*) represents the position of the *i*th atom at time *t*, and N is the total atom number in the system. The variation of SD is a sensitive parameter to investigate the extent of average atomic movement respect to a reference structure. Figure 6 illustrates the variations of SD values of all element types and average enthalpy during the heating process for the single crystal Nb_20.6_Mo_21.7_Ta_15.6_W_21.1_V_21.0_ RHEA. The melting point is also indicated by the dashed line. When temperatures exceed the melting point at 2940 K, the SD profile of each element type displays an abrupt increase. The insert of Fig. 6 shows all SD profiles at temperatures lower than the melting point, and these profiles are closely matched, indicating the collective dynamical behavior of atoms within the RHEA. SD values of all element types increase with the increasing temperature when atoms undergo thermal vibration at temperatures lower than the melting point of 2940 K. At temperatures higher than or equal to the melting point, atoms have enough kinetic energies to leave their lattice sites, so SD values increase dramatically. The distinct rise of SD profiles at 2940 K also confirms the significant local structural arrangement at the melting point of single crystal Nb_20.6_Mo_21.7_Ta_15.6_W_21.1_V_21.0_ RHEA.

**Figure 7:**
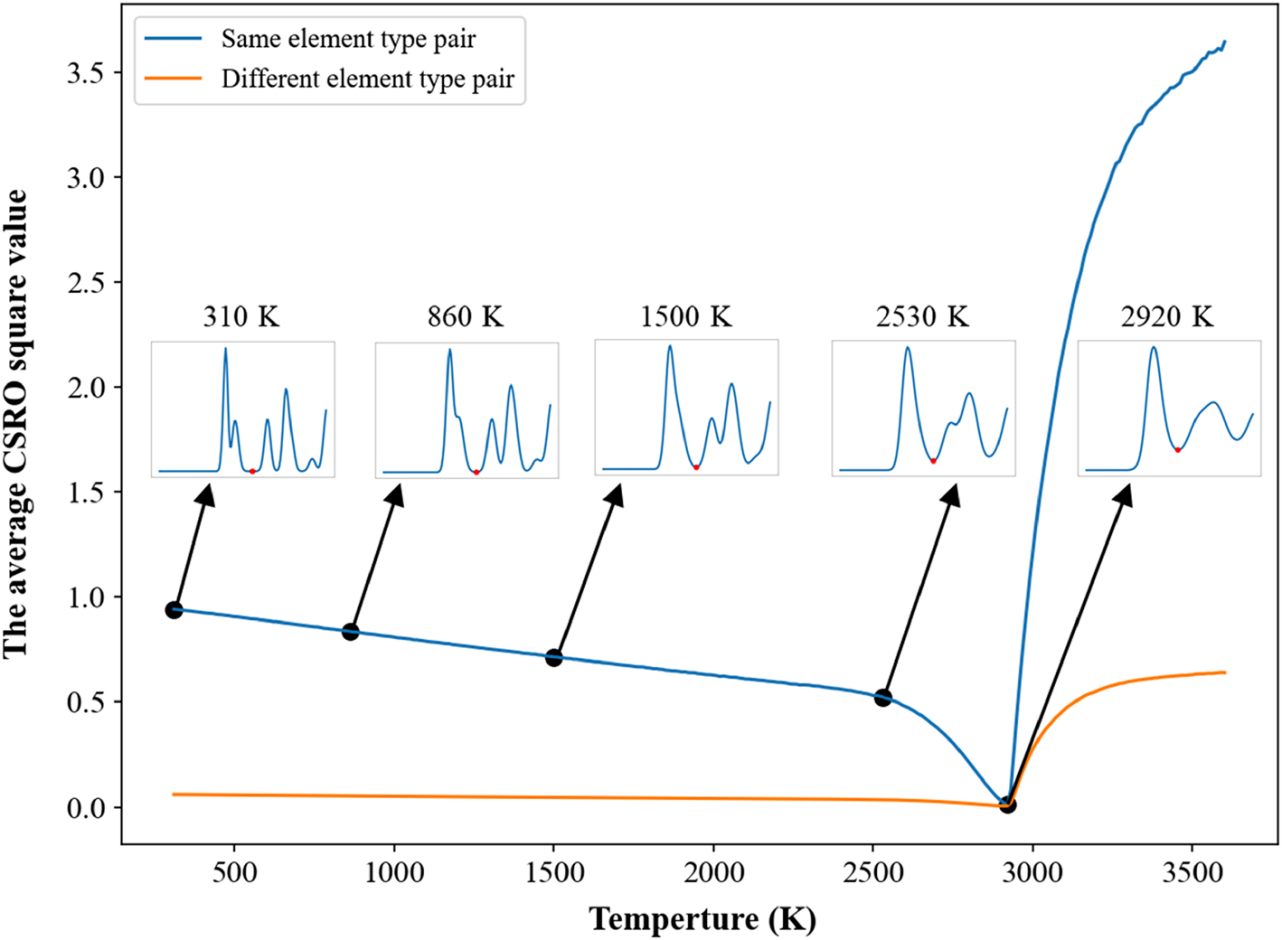
Average CSRO square profiles of the same and different element type pairs for the single crystal Nb_20.6_Mo_21.7_Ta_15.6_W_21.1_V_21.0_ RHEA. The insets show the RDF profiles at different temperatures lower than the melting point of 2940 K.

In Fig. 3 for the single crystal, one can see the short-ranged order values of pairs with the same element type are positive and larger than 0.95, while the short-ranged order values of pairs with different element types are negative. It is very complicated to investigate the short-ranged order values of all pairs during the heating process, so the average values of short-ranged order square for pairs with the same element type and with different element types were used to monitor the change of chemical short-ranged order during the heating process. For the single crystal Nb_20.6_Mo_21.7_Ta_15.6_W_21.1_V_21.0_ RHEA, the average value of short-ranged order square of the same element type shown in Fig. 7 displays linearly decrease with the increasing temperature from 300 to 2530 K, and then decreases parabolically with the rising temperature from 2530 to 2920 K. The extent of lattice distortion and the increase in thermal vibration amplitude become more significant when the system temperature of single crystal Nb_20.6_Mo_21.7_Ta_15.6_W_21.1_V_21.0_ RHEA continuously increases. Consequently, the distance between the first and second RDF peaks becomes closer when the temperature continuously increases, which can be seen in the inserts of RDF profiles at temperatures from 310 to 1500 K. At 1500 K, the first and second RDF peaks have been merged into a single peak. When the temperature continuously increases from 1500 to 2530 K, the first RDF peak becomes wider. It also leads to the linear decrease in the average value of short-ranged order square of the same element type. From 2530 to 2920 K, the value of the first RDF minimum increases with the increasing temperature. Some atoms have higher opportunity to contact with their second and third neighbor atoms with the same element type, leading to the significant decrease in the average short-ranged order square of the same element type from 2530 to 2920 K. At 2920 K, the average short- ranged order square of the same element type reaches its minimum, indicating the chemical short-ranged order of the same element types undergoes a critical change from less affinity to no preference. When the temperature increases from the melting point of 2940 K, the average short-ranged order square value of the same element type significantly rises. For different element types, the average value of short-ranged order square is relatively smaller and remains a constant at temperatures below the melting point of 2940 K. At temperatures above the melting point, the average value of short-ranged order square of different element pairs also displays an abrupt increase, which indicates the short-ranged order of different element types also undergoes a critical change at temperatures above 2940 K.

**Figure 8:**
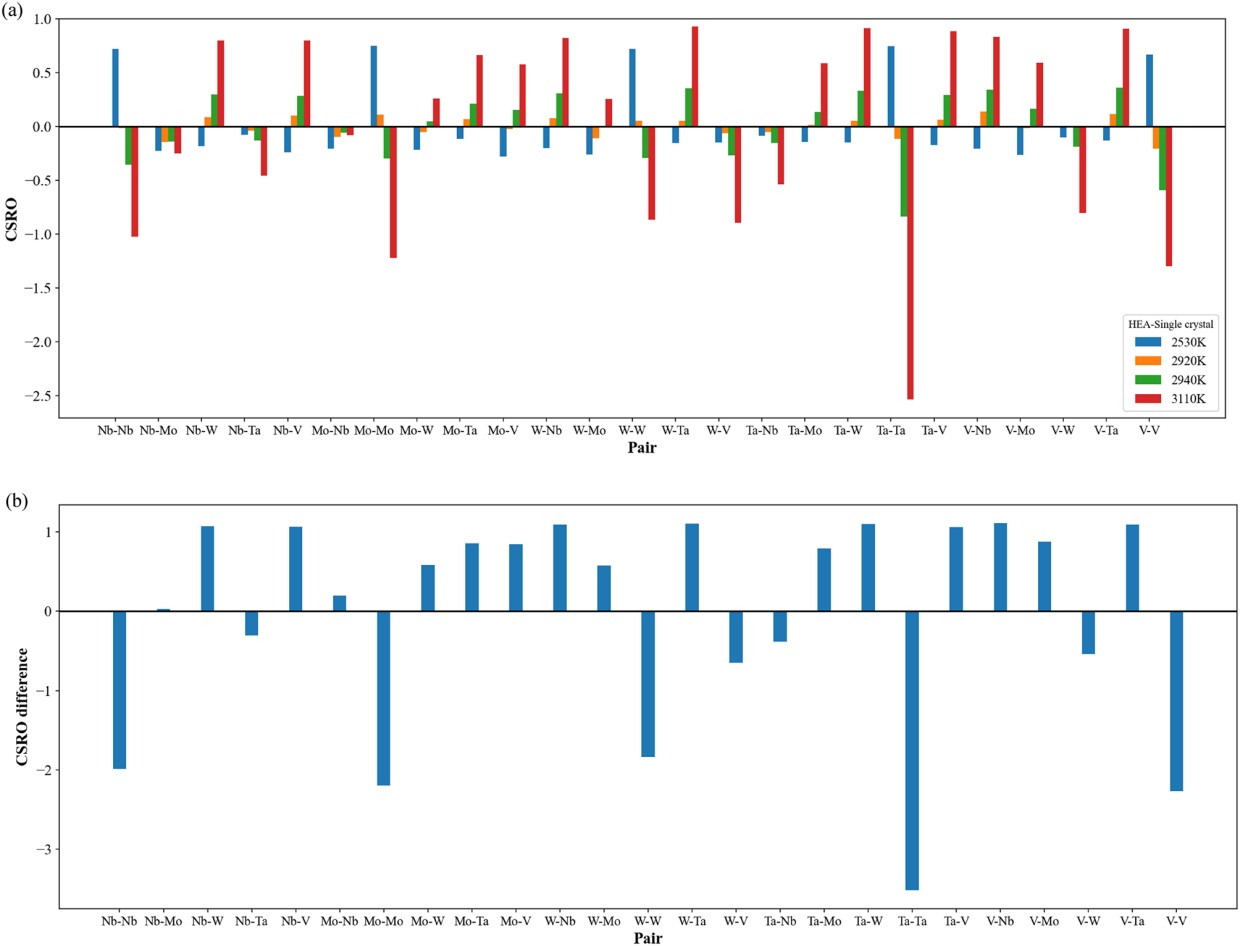
(**a**) The CSRO distributions for single crystal at four characteristic temperatures, 2530, 2920 (minimum of average CSRO square), 2940 (melting point), and 3110 K. (**b**) the differences of CSRO values of all pairs between 300 and 3110 K.

**Figure 9:**
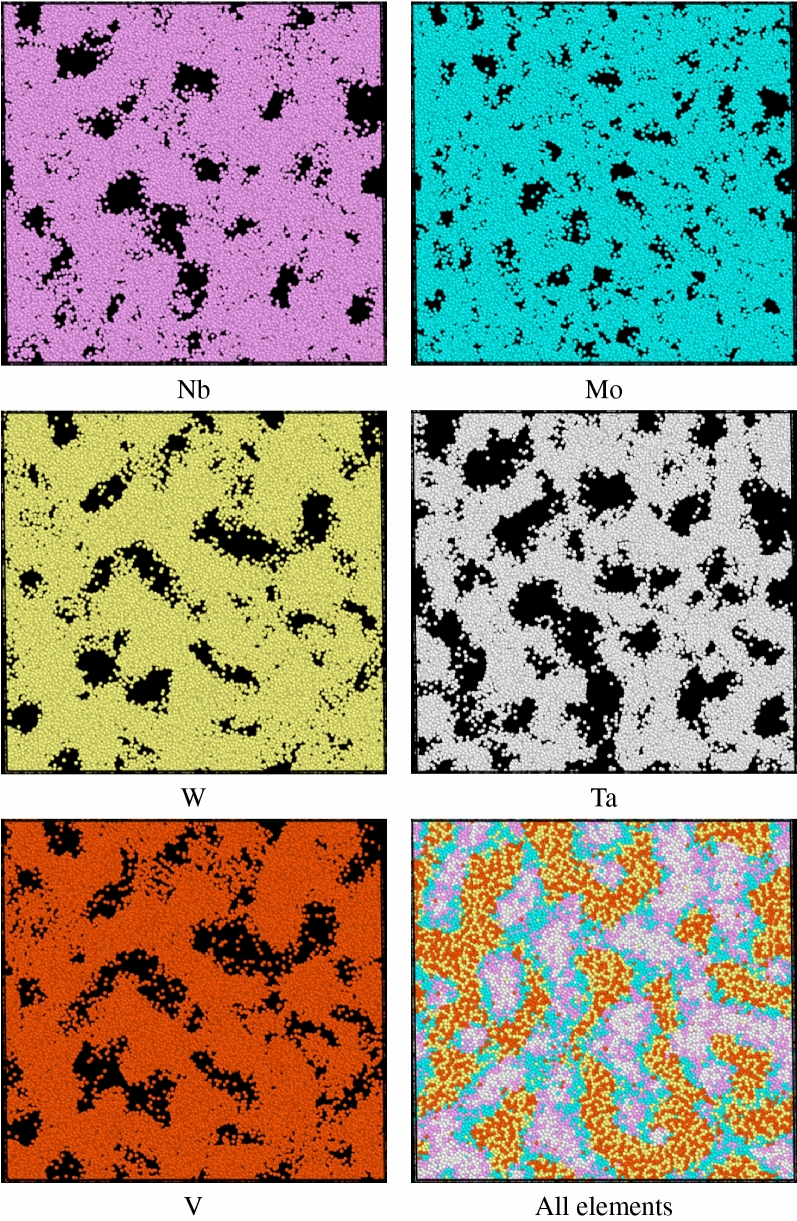
The distributions of Nb, Mo, Ta, W, V, and all elements within the single crystal Nb_20.6_Mo_21.7_Ta_15.6_W_21.1_V_21.0_ RHEA at 3110 K.

Figure 8a demonstrates the short-ranged order distributions of all element type pairs at four characteristic temperatures, 2530, 2920 (minimum of short-ranged order square), 2940 (melting point), and 3110 K. At 2530 K, the short-ranged order values of the same element type pairs are about 0.75, which are smaller than the corresponding values about 0.98 shown in Fig. 3. For the pairs of different element types, the short-ranged order values are very similar to those shown in Fig. 3. At 2920 K, it can be seen from Fig. 8a that the absolute values of all short-ranged order pairs become very small, and the short-ranged order values of the same element pairs are very close to 0. At the melting point of 2940 K and 3110 K, most short-ranged order values show considerable changes as compared to those at 2530 K and those of 300 K shown in Fig. 3. For investigating the short-ranged order change between 300 and 3110 K, Fig. 8b shows the differences of all short-ranged order pairs between 300 and 3110 K. The values in Fig. 8b are the short-ranged order values at 3110 K subtract the corresponding short-ranged order values at 300 K. Consequently, the positive value of short-ranged order difference stands for the chemical affinity of an element type pair becomes weaker, whereas the negative value indicates the affinity of an element type pair becomes stronger. In Fig. 8b, one can see the short-ranged order differences of the same element type pairs are negative, indicating the affinity of the same element undergoes significant change at temperatures higher than the melting point. Figure 9 shows the atom distributions of Nb, Mo, W, Ta, and V within the single crystal Nb_20.6_Mo_21.7_Ta_15.6_W_21.1_V_21.0_ RHEA at 3110 K. One can see the aggregation of Nb, Mo, W, Ta, and V is very obvious. For different element type pairs as shown in Fig. 8b, most of their short-ranged order differences are positive, indicating these element pairs become less affinity after the structural rearrangement after the melting.

**Figure 10:**
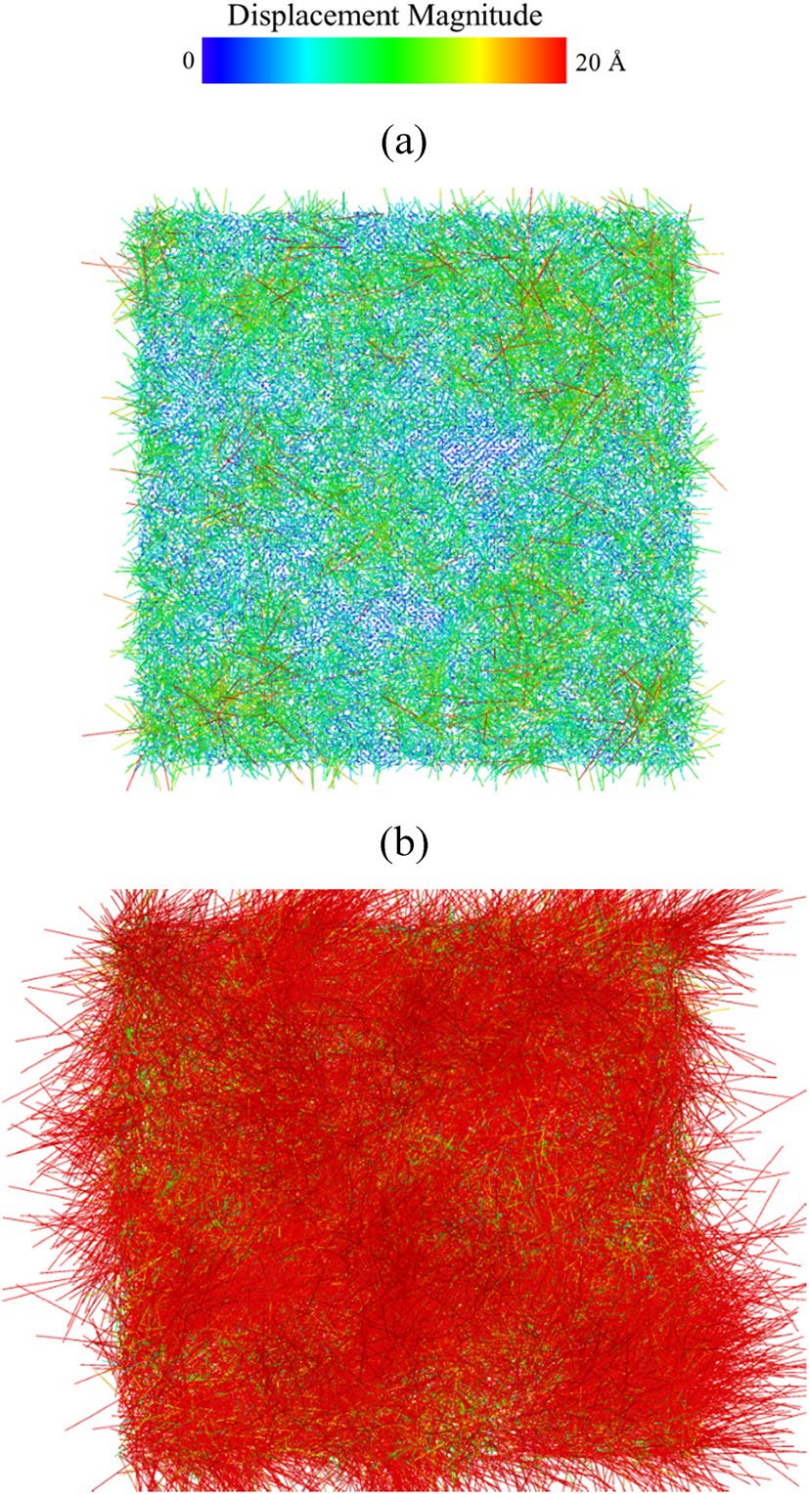
The atomic displacement vectors of the single crystal Nb_20.6_Mo_21.7_Ta_15.6_W_21.1_V_21.0_ RHEA at (**a**) the melting point of 2940 K and (**b**) 3110 K. The atom positions at 300 K were used as the reference positions for calculating the atomic displacement vectors.

Figure 10a,b show the displacement vectors of all atoms at the melting temperature of 2940 K and 3110 K, respectively. The structure at 300 K is used as the reference structure to determine the atomic displacement vectors using OVITO. At 2940 K, one can see the displacement vector lengths of most atoms are longer than those in thermal vibration (marked in blue), indicating these atoms are far away from their equilibrium positions. At 3110 K, the displacement vector lengths of all atoms become much longer and the local structure is under significant change.

**Figure 11:**
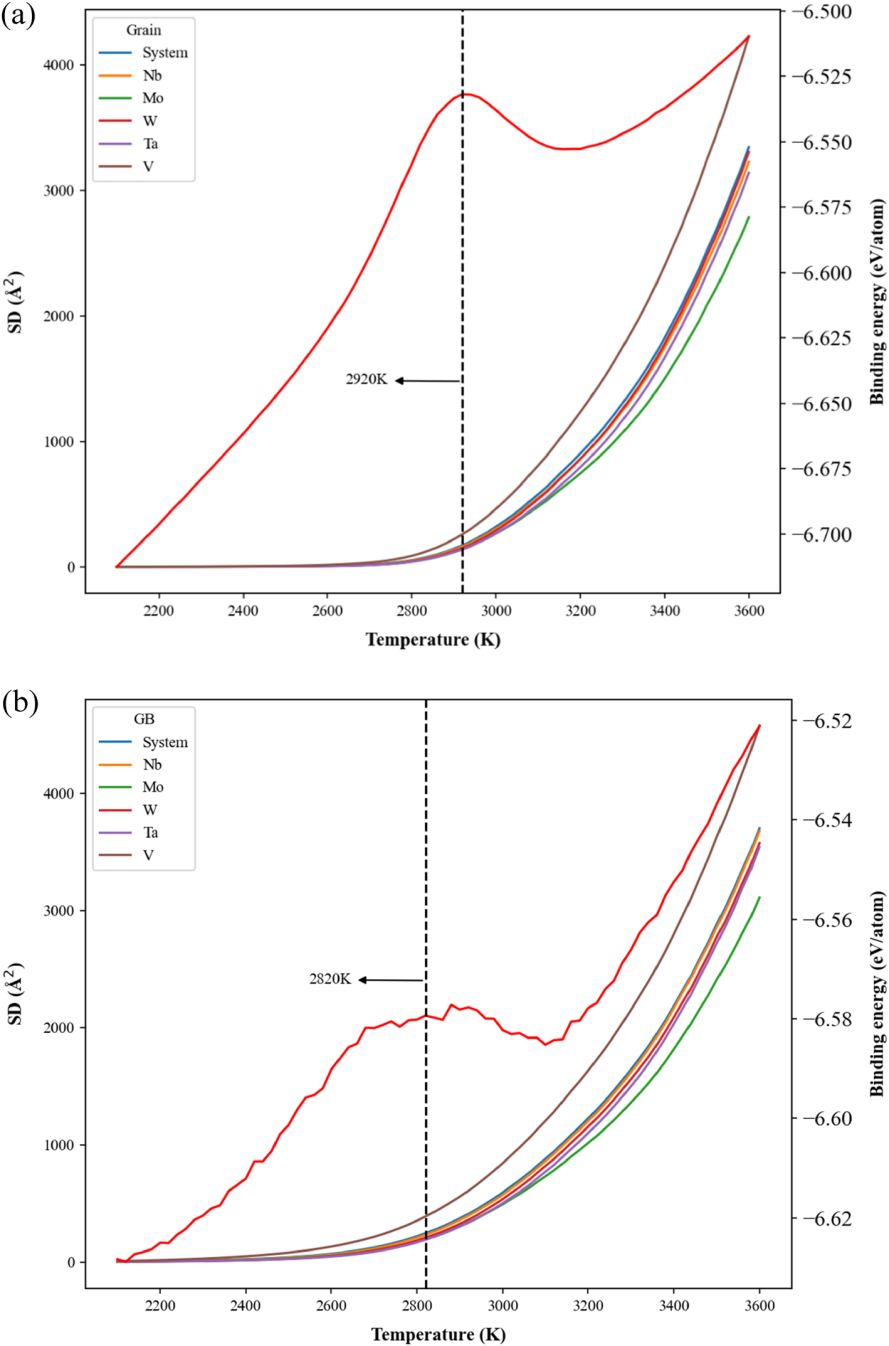
The binding energy and square displacement (SD) profiles of system, Nb, Mo, W, Ta, and V of (**a**) the grain atoms and (**b**) the GB atoms for the case with the average grain size of 25.3 nm during the heating process.

The SD and binding energy profiles of atoms within the grain and GB during the temperature elevation are shown in Fig. 11a,b for the case with the grain size of 25.3 nm, respectively. For the profiles of binding energy, both the values of grain and GB increase linearly with the increasing temperature from 2100 to 2800 K and from 2100 to 2670 K. Then binding energies of grain and GB display parabolic increase from 2800 to 2920 K and from 2670 to 2820 K, respectively. At temperatures higher than 2800 K for grain and 2670 K for GB, SD values begin to rise dramatically, indicating the local structural rearrangement occurs at these temperatures. It can see the temperature for GB structural rearrangement is relatively lower than that of grain. Consequently, at 2820 K, most GB atoms have enough kinetic energies to leave their equilibrium positions, and then these GB atoms gradually induce the rearrangement of grain atoms close to GB. Consequently, the temperature of 2820 K can be regarded as the pre-melting temperature, at which the melting of GB has been completed. When the temperatures continuously increase from 2920 to 3100 K for grain and from 2820 to 3100 K for GB, the binding energies decrease with the increasing temperature, and it indicates atoms of the same element types have a higher opportunity to contact each other. It should be noted the temperature, 2920 K (very close to the melting point obtained from enthalpy profile), is located at the binding energy peak of grain atoms, and it indicates most grain atoms have enough kinetic energies to rearrange. At temperature higher than 3100 K, the binding energies of grain and GB also illustrate linear increase with the increasing temperature.

**Figure 12:**
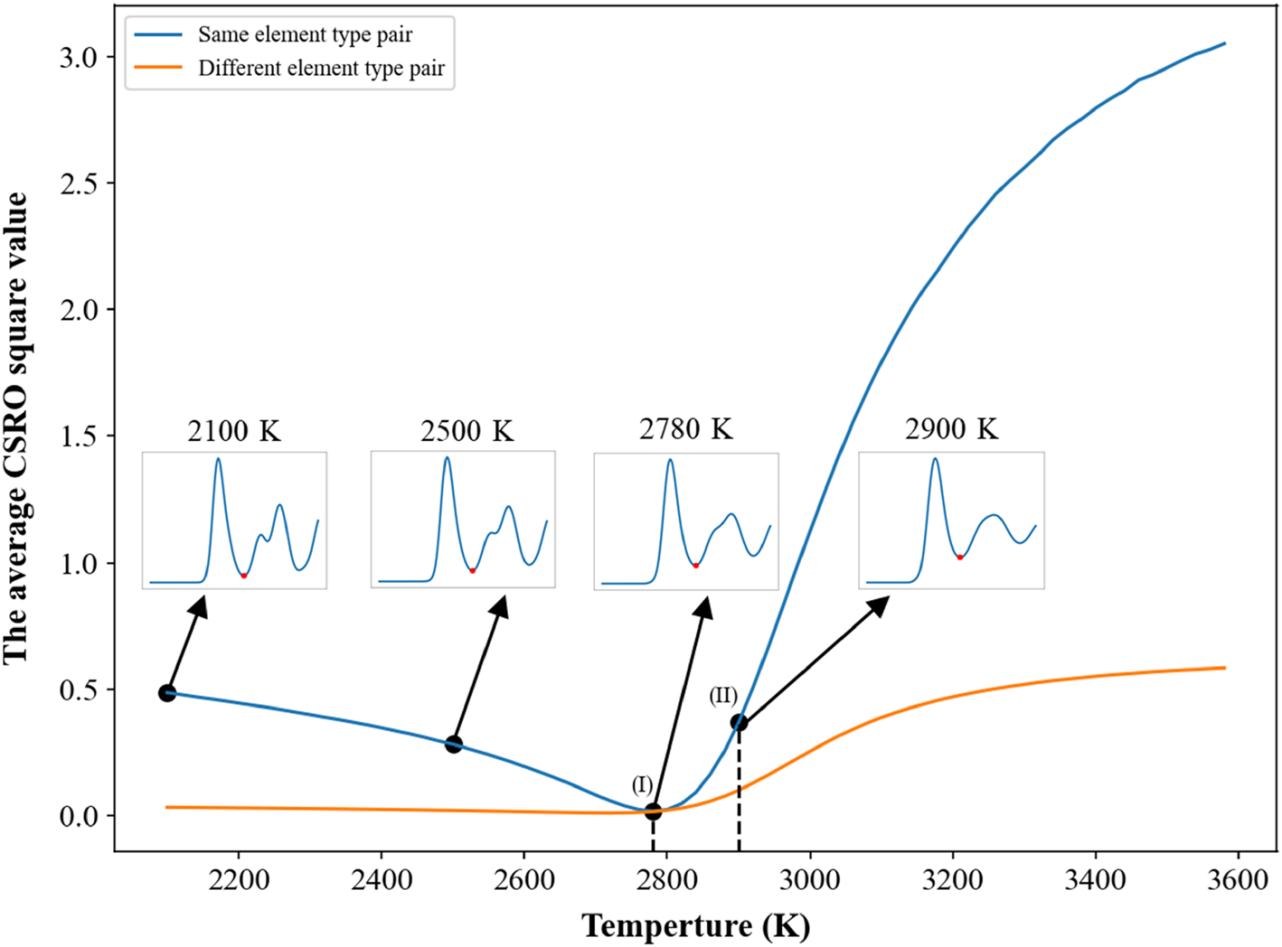
Average CSRO square profiles of the same and different element type pairs for the Nb_20.6_Mo_21.7_Ta_15.6_W_21.1_V_21.0_ RHEA with the average grain size of 25.3 nm. The texts (I) and (II) indicate the lowest value of average CSRO square of the same element pair at 2780 K and the melting point at 2900 K.

**Figure 13:**
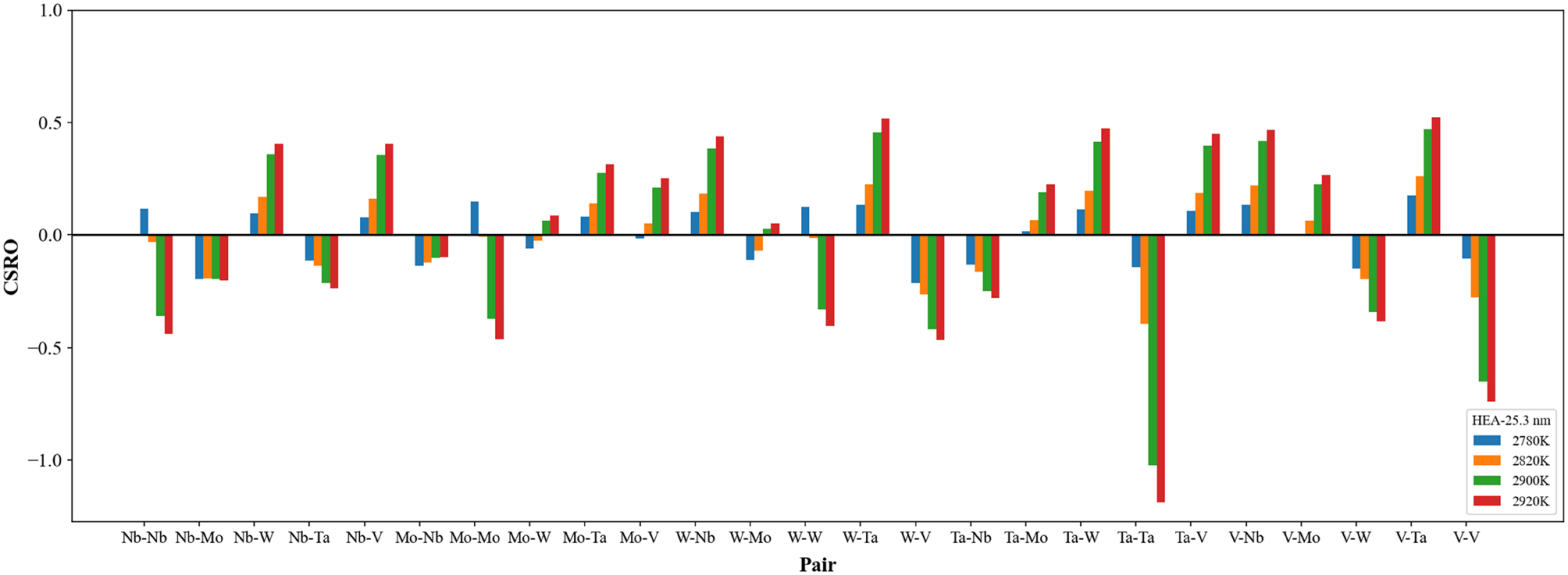
The CSRO distributions for the Nb_20.6_Mo_21.7_Ta_15.6_W_21.1_V_21.0_ RHEA with the average grain size of 25.3 nm at four characteristic temperatures, 2780 (minimum of average CSRO square), 2820 (pre-melting temperature), 2900 (melting point), and 2920 (binding energy peak of grain atom) K.

The average short-ranged order square profiles of the case with the average grain size of 25.3 nm during the heating process were shown in Fig. 12. RDF profiles at different temperatures are also shown in the inserts. One can see the value of the first RDF minimum become larger when the temperature continuously increases, leading to the decrease in the average short-ranged order square value. The minimum of the average short-ranged order square is at 2780 K as indicated by (I), below which the average short-ranged order square value of different element pairs is almost constant. When the temperature continuously increases from 2780 K to the melting point of 2900 K, both short-ranged order square profiles display linear increase with the increasing temperature. When the temperature increases from the melting point, these two short-ranged order square profiles begin the significant increase with the increasing temperature. The short-ranged order distributions for the Nb_20.6_Mo_21.7_Ta_15.6_W_21.1_V_21.0_ RHEA with the average grain size of 25.3 nm at 2780 (minimum of average short-ranged order square), 2820 (pre-melting temperature), 2900 (melting point), and 2920 (binding energy peak of grain atom) K are illustrated in Fig. 13. At the pre-melting temperature, 2820 K, the GB atoms and some grain atoms close to GB have undergone significant local structural arrangement, leading to the short-ranged order changes of these atoms. The short-ranged order value variations of different element pairs at temperatures higher than the pre-melting temperature are very similar to those of single crystal Nb_20.6_Mo_21.7_Ta_15.6_W_21.1_V_21.0_ RHEA at temperatures higher than that with the minimum of average short-ranged order square as shown in Fig. 8a.

**Figure 14:**
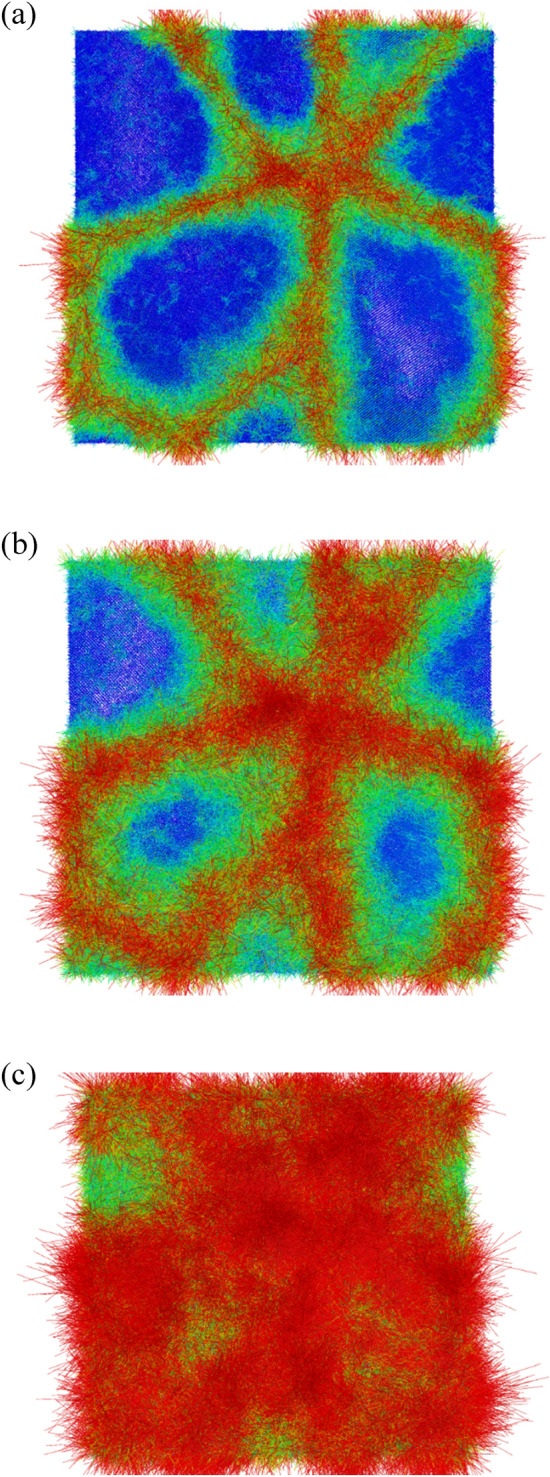
The atomic displacement vectors of 25.3 nm at (**a**) 2820 K, (**b**) 2920 K, and (**c**) 3100 K, respectively. The atom positions at 300 K were used as the reference positions for the displacement vectors.

For investigating the melting processes of GB and grain of the Nb_20.6_Mo_21.7_Ta_15.6_W_21.1_V_21.0_ RHEA with the average grain size of 25.3 nm, the atom displacement vectors of 2820 (the pre-melting temperature), 2920 (20 K higher than the melting point), and 3100 K shown in Fig. 14a–c were used. The atom positions of the structure at 300 K were used as the reference, and the vectors were colored according to the length of a vector. In Fig. 14a, most GB atoms have larger displacement vector sizes (marked in red), and some grain atoms close to the GB atoms are also affected by the GB atoms, which possess larger displacement vector sizes (marked in green) as compared to those at the cores of grains (marked in blue). At 2920 K as shown in Fig. 14b, more grain atoms have large displacement vectors and the melting occurs toward the cores of grains. At 3100 K, the displacement vectors in Fig. 14c indicates all atoms within the Nb_20.6_Mo_21.7_Ta_15.6_W_21.1_V_21.0_ RHEA with the average grain size of 25.3 nm have left their equilibrium positions and the system is in the melting state.

**Figure 15:**
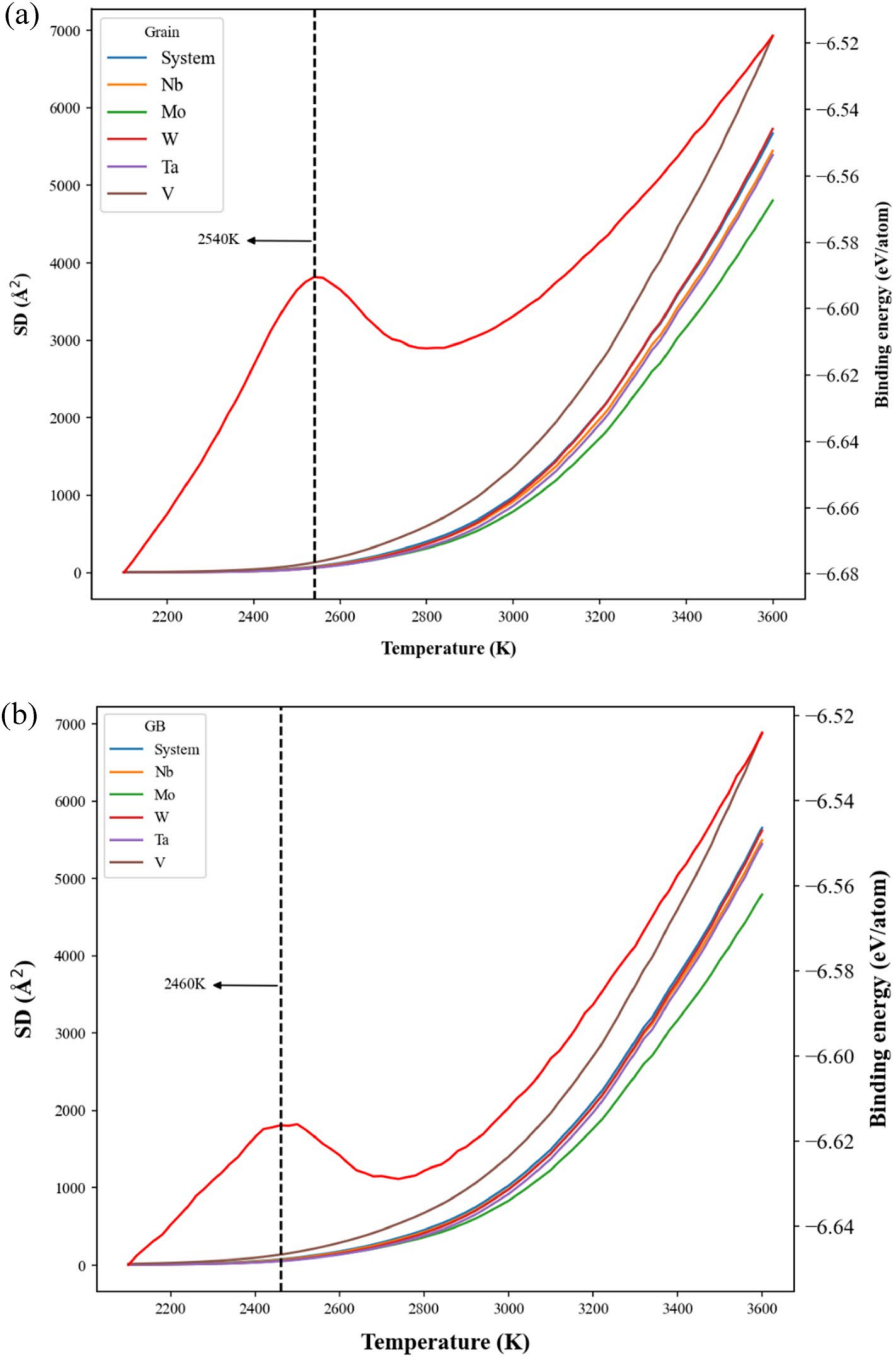
The binding energy and square displacement (SD) profiles of system, Nb, Mo, W, Ta, and V of (**a**) the grain atoms and (**b**) the GB atoms for the case with the average grain size of 5.2 nm during the heating process.

The SD and binding energy profiles of grain and GB atoms during the temperature elevation process are shown in Fig. 15a,b for the case with the average grain size of 5.2 nm, respectively. Variations of SD and binding energies with the increasing temperature are very similar to those of the case with the grain size of 25.3 nm as shown in Fig. 11a,b. For the case with a smaller average grain size, the pre-melting temperature of 2460 K is lower than that of 25.3 nm about 2820 K.

**Figure 16:**
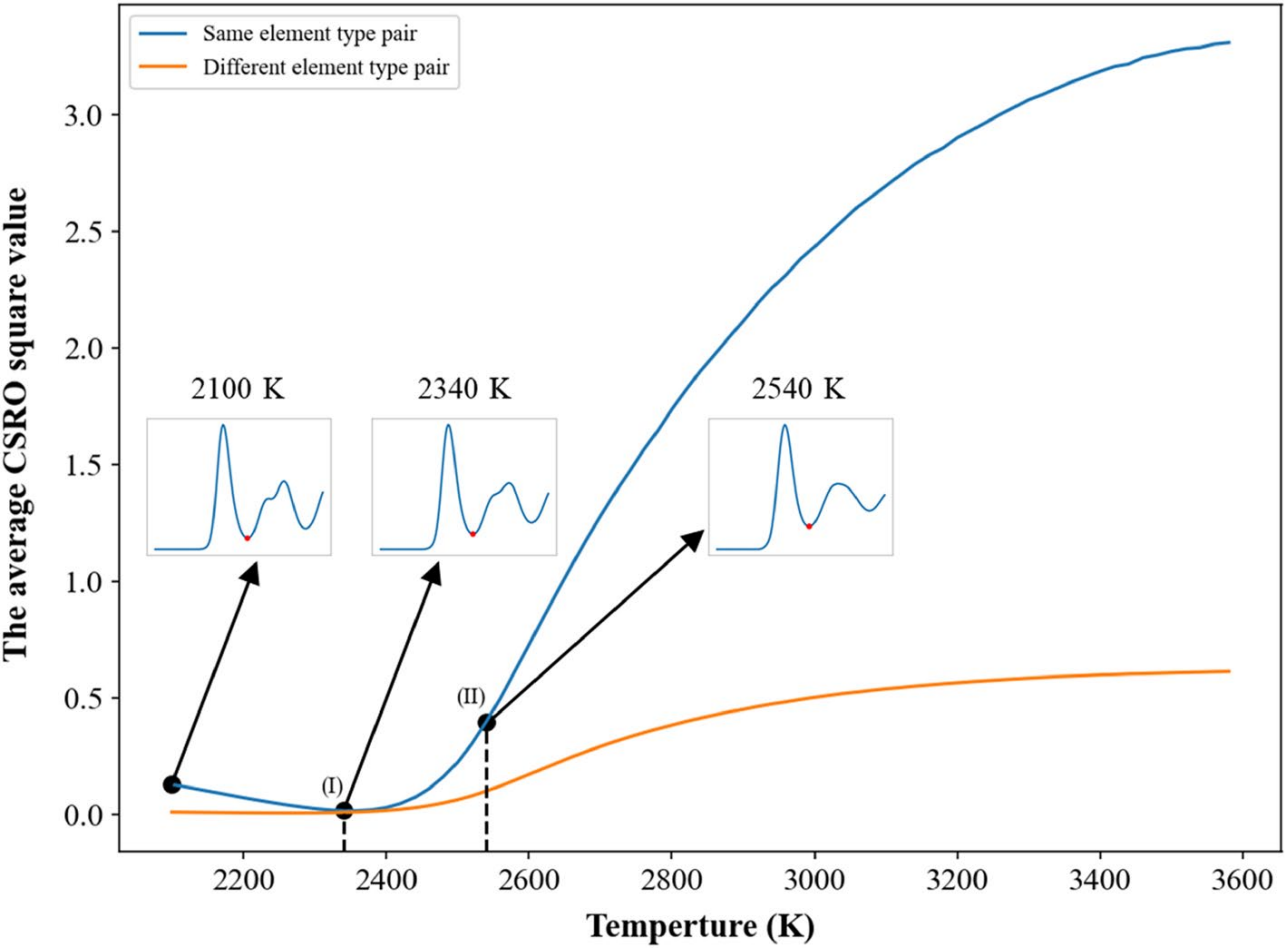
Average CSRO square profiles of the same and different element type pairs for the Nb_20.6_Mo_21.7_Ta_15.6_W_21.1_V_21.0_ RHEA with the average grain size of 5.2 nm. The texts (I) and (II) indicate the minimum of average CSRO square of the same element pair at 2340 K and the melting point at 2540 K.

**Figure 17:**
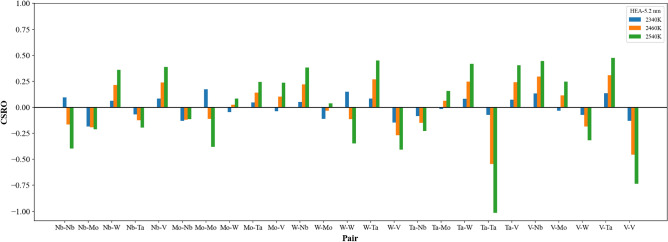
The CSRO distributions for the Nb_20.6_Mo_21.7_Ta_15.6_W_21.1_V_21.0_ RHEA with the average grain size of 5.2 nm at 2340 (minimum of average CSRO square), 2460 (pre-melting temperature), and 2540 (melting point and the binding energy peak of grain atom) K.

The average short-ranged order square profiles of the case with the average grain size of 5.2 nm during the heating process were shown in Fig. 16. RDF profiles at different temperatures are also shown in the inserts. The value of the first RDF minimum also becomes larger when the temperature continuously increases, leading to the decrease in the average short-ranged order square value. The minimum of the average short-ranged order square is at 2340 K as indicated by (I), below which the average short-ranged order square value of different element pairs is almost constant. When the temperature continuously increases from 2340 K to the pre-melting temperature of 2460 K, both short-ranged order square profiles slightly increase with the increasing temperature. Within this temperature range, most GB atoms have enough kinetic energies to rearrange, while most grain atoms still conduct the thermal vibration at their equilibrium positions. From the pre-melting temperature of 2460 K to the melting temperature of 2540 K, GB atoms leaving their equilibrium positions further affect grain atoms close to GB atoms to leave from their equilibrium positions. Consequently, these two short-ranged order square profiles increase significantly with the increasing temperature, revealing the short-ranged order of this RHEA undergoes considerable change. The short-ranged order distributions for the Nb_20.6_Mo_21.7_Ta_15.6_W_21.1_V_21.0_ RHEA with the average grain size of 5.2 nm at 2340 (minimum of short-ranged order square), 2460 (pre-melting temperature), and 2540 (melting point and the binding energy peak of grain atom) K are illustrated in Fig. 17. Short-ranged order variations of different element pairs during the melting process are very similar to those shown in Fig. 13 for the case of 25.3 nm.

**Figure 18:**
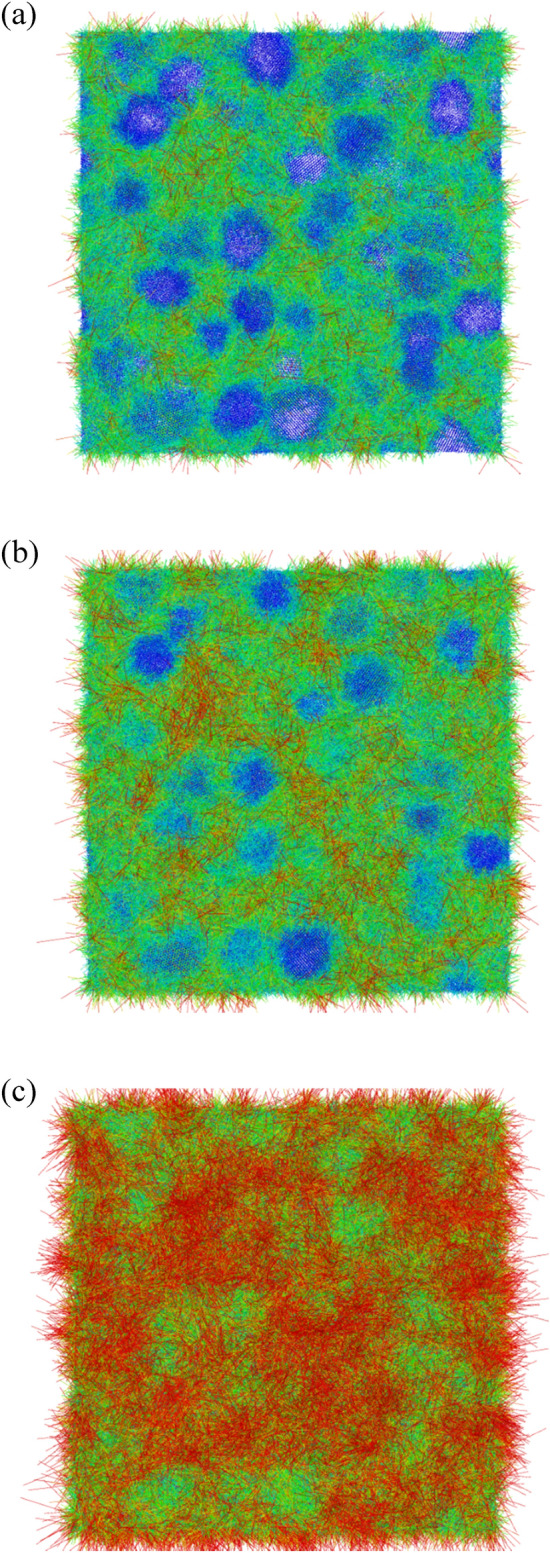
The atomic displacement vectors of the case with the grain size of 5.2 nm at (**a**) 2460 K (pre-melting temperature), (**b**) 2540 K (melting point and the binding energy peak of grain atom), and (**c**) 2700 K, respectively. The atom positions at 300 K were used as the reference positions for the displacement vectors.

The atom displacement vectors at 2460 K (pre-melting temperature), 2540 K (melting point and the binding energy peak of grain atom), and 2700 K are shown in Fig. 18a–c. The structure at 2000 K was used as the reference for calculating the atomic displacement vectors. The atom positions of the structure at 300 K were used as the reference, and the vectors were colored according to the length of a vector. In Fig. 18a, GB atoms and grain atoms close to GB have longer displacement vector sizes (marked in red and green), as compared with those at the cores of grains (marked in blue). At 2540 K as shown in Fig. 18b, more grain atoms have large displacement vector lengths and the melting occurs toward the cores of grains. At 2700 K, the displacement vectors in Fig. 18c indicates all atoms within the Nb_20.6_Mo_21.7_Ta_15.6_W_21.1_V_21.0_ RHEA with the average grain size of 5.2 nm leave their equilibrium positions and the system is in the melting state.

## Conclusion

This study employs the MD simulation using 2NN MEAM potential to investigate the melting mechanism and the short-ranged order change for the single crystal and polycrystalline Nb_20.6_Mo_21.7_Ta_15.6_W_21.1_V_21.0_ RHEAs with the average grain sizes of 5.2, 10.0, 15.6, 20.1, and 25.3 nm. The MaxEnt theory was used to build the structures for all Nb_20.6_Mo_21.7_Ta_15.6_W_21.1_V_21.0_ RHEAs. For polycrystalline Nb_20.6_Mo_21.7_Ta_15.6_W_21.1_V_21.0_ RHEAs, the GB atom fraction increases parabolically from 6.2 to 26.8% when the average grain size decreases from 25.3 to 5.2 nm. At 300 K, short-ranged order values of the same element type pairs are larger than 0.8, indicating that all element types are arranged in the most uniform distribution. For pairs of different element types, all short-ranged order values are smaller than − 0.1, indicating that the affinity of these pairs is much higher than those with the same element types. During the heating process, the density profile of single crystal Nb_20.6_Mo_21.7_Ta_15.6_W_21.1_V_21.0_ RHEA displays an abrupt drop from 11.25 to 11.00 g/cm^3^ from 2910 to 2940 K, within which the enthalpy reaches its maximum value. It indicates all atoms begin the significant local structural rearrangement at 2940 K, implying the atoms of single crystal Nb_20.6_Mo_21.7_Ta_15.6_W_21.1_V_21.0_ RHEA have a collective behavior during the melting process. When the temperature increases from 2940 K, the enthalpy first becomes lower until 3110 K and then displays linear increase with the increasing temperature from 3110 K. When the system temperature is higher than the melting point of 2940 K, atoms have enough kinetic energies to leave their equilibrium positions, and the elements possessing higher binding energies begin to aggregate, resulting in the decrease of enthalpy from 2940 to 3110 K.

For the melting mechanism of polycrystalline Nb_20.6_Mo_21.7_Ta_15.6_W_21.1_V_21.0_ RHEAs, a two-stage melting process is proposed. The first melting stage is the melting of GB, and then the second melting stage is the melting of grains. In the first melting stage, the temperature for GB structural rearrangement is relatively lower than that of grain, and this temperature is the pre-melting temperature, at which most GB atoms have enough kinetic energies to leave their equilibrium positions, and then these GB atoms gradually induce the rearrangement of grain atoms close to GB. Pre-melting temperatures of grains of 25.3 nm and 5.2 nm are 2820 K and 2460 K, inferring the pre-melting temperature significantly depends on the average grain size. In the second melting stage at the melting point, most grain atoms have enough kinetic energies to rearrange, resulting in the short-ranged order changes of all pairs.

The CNA analysis results clearly indicate the local structures of GB atoms are amorphous with the undefined type of CNA result, and the fraction of GB atoms decreases with the increasing grain size. The average binding energy of amorphous structure is higher than that of crystal one within the grains. Consequently, the binding energy decreases with the increasing the grain size, resulting in a higher melting point. According to the melting points obtained by the MD simulation, the melting point and the grain size of polycrystalline Nb_20.6_Mo_21.7_Ta_15.6_W_21.1_V_21.0_ RHEAs have the relationship of logarithmic growth. The melting point, T_m_(d), of the Nb_20.6_Mo_21.7_Ta_15.6_W_21.1_V_21.0_ RHEA with the average grain size of *d* nm can be determined by the following formula:

4 $${T}_{m}\left(d\right)=2940 \times Exp\left(-\frac{1.2}{{d}^{1.27}}\right).$$

For the short-ranged order difference between 300 K and the melting point, the cases of single crystal and polycrystalline Nb_20.6_Mo_21.7_Ta_15.6_W_21.1_V_21.0_ RHEAs are very similar. Short-ranged order differences of the same element type pairs are negative, indicating the affinity between the same element types undergoes significant change at temperatures higher than the melting point, resulting in the aggregation of Nb, Mo, W, Ta, and V. For different element type pairs, most of their short-ranged order differences are positive, implying that these element type pairs become less affinity after the structural rearrangement at temperatures higher than the melting point.

### Simulation model

The second-nearest neighbor modified embedded atomic method (2NN MEAM) was used to describe the interactions among Nb, Mo, T, W, and V atoms. Table 1 lists the parameters of all single elements^32^, and Tables 2 and 3 show all cross-element and ternary-element parameters of 2NN MEAM potential, parametrized by the reference data prepared by the density functional theory (DFT) calculation. The CASTEP package was used for all DFT calculations, and the generalized gradient approximation (GGA) with the parameterization of RPBE was used^33^. For the electronic step, the energy tolerance in the self-consistent field calculation was 1.0 × 10^–6^ eV. For the ionic step, the energy, force, and atomic displacement tolerance were 1.0 × 10^–5^ eV, 3.0 × 10^–2^ eV/Å, and 1.0 × 10^–3^ Å, respectively. To obtain the cross-element 2NN MEAM parameters, the B2 structures of NbMo, NbTa, NbW, NbV, MoTa, MoW, MoV, TaW, TaV, and WV element pairs were used as the reference structures in the DFT calculations for the parametrization of 2NN MEAM potential. Besides the binding energies of B2 structures, the binding energies of the following reference structures were also used for the parametrization process:

1. The binding energies of all B2 structures having one atom void. The binding energies of (100), (110), and (111) surfaces as well as the generalized stacking fault energies (GSFE) for all B2 structures.
2. The Nb-Mo-Ta-W-V HEA structure optimized by the DFT calculation.
3. Using the optimized Nb-Mo-Ta-W-V HEA, the structures of the corresponding GSFE profiles for the (112) plane along [11-1] direction, the (110) plane along [010] direction, the (111) plane along [1–10] direction, and (112) plane along [11-1] direction.
4. The binding energies of structures generated by the molecular dynamics simulation at the NPT ensemble. The MD simulations were conducted for 100 steps with a time step of 2.5 fs, and the temperatures of 300, 600, and 900 K were considered at 0 GPa for all cases.

**Table 1:** The 2NN MEAM parameters for Nb, Mo, T, W, and V atoms.

	E_C_	r_e_	$B$	$A$	${\beta }^{(0)}$	${\beta }^{(1)}$	${\beta }^{(2)}$	${\beta }^{(3)}$	${t}^{(1)}$	${t}^{(2)}$	${t}^{(3)}$	C_max_	C_min_	d
Nb	7.47	2.860	1.73	0.72	5.08	1.0	2.5	1.0	1.7	2.8	− 1.6	2.80	0.36	0.00
Mo	6.81	2.725	2.65	0.46	7.03	1.0	1.0	1.0	0.5	3.1	− 7.5	2.80	0.64	0.00
Ta	8.09	2.860	1.94	0.67	4.49	1.0	1.0	1.0	1.7	2.1	− 3.2	2.80	0.25	0.00
W	8.66	2.740	3.14	0.40	6.54	1.0	1.0	1.0	− 0.6	0.3	− 8.7	2.80	0.49	0.00
V	5.30	2.625	1.57	0.73	4.74	1.0	2.5	1.0	3.3	3.2	− 2.0	2.80	0.49	0.00

**Table 2:** The cross-element parameters of 2NN MEAM potential for Nb, Mo, T, W, and V atoms in the LAMMPS format.

Reference	NbMo	NbW	NbTa	NbV	MoW	MoTa	MoV	WTa	WV	TaV
	B2	B2	B2	B2	B2	B2	B2	B2	B2	B2
Ec	7.201	8.082	7.758	6.274	7.737	7.380	6.076	8.277	7.001	6.616
r_e_	2.806	2.816	2.904	2.761	2.752	2.831	2.673	2.839	2.682	2.781
α	5.760	5.576	5.222	4.890	5.715	6.087	5.870	5.696	5.508	5.251
Attract	− 0.100	− 0.100	− 0.007	− 0.100	− 0.050	− 0.100	− 0.100	− 0.100	0.100	− 0.100
Repuls	0.011	0.010	0.062	0.035	0.080	0.005	0.002	0.025	0.100	0.054
${\rho }_{0}^{A}/{\rho }_{0}^{B}$	1.000	1.000	1.000	1.000	1.000	1.000	1.000	1.000	1.000	1.000
C_min_ (A-B-A)	0.102	0.809	0.933	0.281	0.100	0.990	0.455	0.737	0.100	0.630
C_min_ (B-B-A)	0.100	0.125	0.100	0.211	0.651	0.455	0.578	0.639	0.629	0.583
C_min_ (A-A-B)	0.100	0.753	0.217	0.625	0.408	0.411	0.528	0.269	0.100	0.100
C_min_ (A-B-B)	0.990	0.434	0.153	0.990	0.100	0.990	0.254	0.990	0.100	0.955
C_max_ (A-B-A)	2.000	2.438	2.463	2.454	2.000	2.800	2.634	2.799	2.000	2.190
C_max_ (B-B-A)	2.102	2.231	2.130	2.507	2.154	2.205	2.245	2.107	2.230	2.228
C_max_ (A-A-B)	2.272	2.257	2.355	2.033	2.199	2.234	2.162	2.072	2.000	2.272

**Table 3:** The parameter sets of C_min_ and C_max_ for ternary elements in the LAMMPS format.

		C_min_	C_max_
Nb-Mo-W	(Nb-Mo-W)	0.652	2.565
	(Nb-W-Mo)	0.584	2.192
	(Mo-W-Nb)	0.990	2.800
Nb-Mo-Ta	(Nb-Mo-Ta)	0.425	2.303
	(Nb-Ta-Mo)	0.990	2.409
	(Mo-Ta-Nb)	0.100	2.676
Nb-Mo-V	(Nb-Mo-V)	0.694	2.712
	(Nb-V-Mo)	0.561	2.800
	(Mo-V-Nb)	0.685	2.167
Nb-W-Ta	(Nb-W-Ta)	0.100	2.513
	(Nb-Ta-W)	0.985	2.487
	(W-Ta-Nb)	0.990	2.800
Nb-W-V	(Nb-W-V)	0.693	2.477
	(Nb-V-W)	0.915	2.633
	(W-V-Nb)	0.778	2.389
Nb-Ta-V	(Nb-Ta-V)	0.752	2.365
	(Nb-V-Ta)	0.693	2.233
	(Ta-V-Nb)	0.657	2.698
Mo-W-Ta	(Mo-W-Ta)	0.801	2.512
	(Mo-Ta-W)	0.257	2.800
	(W-Ta-Mo)	0.990	2.800
Mo-W-V	(Mo-W-V)	0.990	2.800
	(Mo-V-W)	0.858	2.077
	(W-V-Mo)	0.479	2.080
Mo-Ta-V	(Mo-Ta-V)	0.771	2.254
	(Mo-V-Ta)	0.368	2.176
	(Ta-V-Mo)	0.364	2.681
W-Ta-V	(W-Ta-V)	0.990	2.578
	(W-V-Ta)	0.785	2.778
	(Ta-V-W)	0.763	2.800

With the fitted cross-element parameters listed in Tables 2 and 3, the errors between the energies by the DFT calculation and 2NN MEAM potential of all Nb_20.6_Mo_21.7_Ta_15.6_W_21.1_V_21.0_ RHEA reference structures (20 at respective temperatures of 300, 600, and 900 K) are within − 5% ~  + 5%. In addition, a MD simulation of the BCC single crystal Nb_20.6_Mo_21.7_Ta_15.6_W_21.1_V_21.0_ RHEA at the isothermal-isobaric (NPT) ensemble (300 K and 0 GPa) was conducted for 50 ps. The structure of BCC single crystal Nb_20.6_Mo_21.7_Ta_15.6_W_21.1_V_21.0_ RHEA maintains the 100% local BCC structure. The predicted density is about 11.9 g/cm^3^, which is very close to the related experimental value of 12.3 g/cm^3^^16^. Furthermore, the predicted melting temperature of single crystal Nb_20.6_Mo_21.7_Ta_15.6_W_21.1_V_21.0_ RHEA using current 2NN MEAM parameters is 2940 K, which is also very close to the predicted value about 2946 K in Senko’s study^31^. Consequently, it indicates the cross-element parameters of 2NN MEAM potentials listed in Tables 2 and 3 are liable for Nb-Mo-Ta-W-V systems.

The ATOMSK package^34^ was adopted to build the structures of polycrystalline Nb_20.6_Mo_21.7_Ta_15.6_W_21.1_V_21.0_ RHEAs with the average grain sizes of 25.3, 20.1, 15.6, 10.0, and 5.2 nm. Figure 1 shows the polycrystalline Nb_20.6_Mo_21.7_Ta_15.6_W_21.1_V_21.0_ RHEA with the average grain size of 5.2 nm. The system size is about 40.7, 40.7, and 40.7 nm in the x-, y-, and z-dimensions, respectively. The atoms in Fig. 1a–c are colored according to the element type, grain and grain boundary atoms identified by the common neighbor analysis (CNA)^35^, and the grain identity number, respectively. Table 4 summarize the grain number and total atom number of the Nb_20.6_Mo_21.7_Ta_15.6_W_21.1_V_21.0_ RHEAs with average grain sizes from 5.2 to 31.9 nm. The total atom number is about 4,113,600, and the atom numbers of different cases are slightly different. The single crystal Nb_20.6_Mo_21.7_Ta_15.6_W_21.1_V_21.0_ RHEA with 1,024,000 atoms is also listed in Table 4.

**Table 4:** The models of single crystal and polycrystalline Nb_20.6_Mo_21.7_Ta_15.6_W_21.1_V_21.0_ RHEAs for the heating simulation.

Grain size (nm)	Grain number	Atom number
Single crystal	X	1,024,000
25.3	4	4,113,488
20.1	8	4,112,402
15.6	17	4,113,753
10.0	65	4,113,509
5.2	450	4,113,688

The maximum entropy (MaxEnt) theory^36^ implemented by Monte Carlo (MC) method was used to have each compositional element undergo the most uniform distribution within all Nb_20.6_Mo_21.7_Ta_15.6_W_21.1_V_21.0_ RHEAs, resulting in the maximum configurational entropy state. The element type of each nearest neighbor atom of a reference atom is not the same as the element type of the reference atom. Because all atoms were arranged by the MaxEnt theory, each element type is in its most uniform distribution throughout all Nb_20.6_Mo_21.7_Ta_15.6_W_21.1_V_21.0_ RHEA cases.

Thermal behaviors of single crystal and polycrystalline Nb_20.6_Mo_21.7_Ta_15.6_W_21.1_V_21.0_ RHEAs were investigated by the MD temperature elevation process from 300 to 3600 K. The heating process was processed in the increasing temperature by 10 K increment, and each increment was accompanied by a relaxation process in 10 ps before the subsequent temperature increases. For maintaining the constant temperature under the free stress during the temperature elevation process, the TtN method was utilized^37^. This method combines the Parrinello–Rahman variable shape size ensemble with the Nosé–Hoover thermostat. For the heating simulation, the periodic boundary conditions (PBCs) were used in all dimensions. Large-scale atomic/molecular massively parallel simulator (LAMMPS) was utilized to perform all MD simulations, which was developed by Plimpton et al*.*^38^. The OVITO package^39^ was used to do all visualization and post process of all simulation results.

## References

[1] Nie XW, Cai MD, Cai S (2021). Microstructure and mechanical properties of a novel refractory high entropy alloy HfMoScTaZr. Int. J. Refract. Met. Hard Mater..

[2] Taleghani PR (2014). Phase and microstructural characterization of Mo–Si–B multiphase intermetallic alloys produced by pressureless sintering. J. Alloy. Compd..

[3] Bhandari U, Zhang C, Yang S (2020). Mechanical and thermal properties of low-density Al_20+x_Cr_20-x_Mo_20-y_Ti_20_V_20+y_ alloys. Curr. Comput.-Aided Drug Des..

[4] Gao XJ (2021). Microstructure characteristics and mechanical properties of Hf_0.5_Mo_0.5_NbTiZr refractory high entropy alloy with Cr addition. Int. J. Refract. Met. Hard Mater..

[5] Sun SJ (2021). Revisiting the role of prestrain history in the mechanical properties of ultrafine-grained CoCrFeMnNi high-entropy alloy. Mater. Sci. Eng. A.

[6] Huo W (2018). Ultrahigh hardness and high electrical resistivity in nano-twinned, nanocrystalline high-entropy alloy films. Appl. Surf. Sci..

[7] Sun SJ (2017). Enhanced strength and ductility of bulk CoCrFeMnNi high entropy alloy having fully recrystallized ultrafine-grained structure. Mater. Des..

[8] Li Z (2016). Metastable high-entropy dual-phase alloys overcome the strength—ductility trade-off. Nature.

[9] Tang Z (2015). Fatigue behavior of a wrought Al_0.5_CoCrCuFeNi two-phase high- entropy alloy. Acta Mater..

[10] Yang X (2020). A novel, non-equiatomic NiCrWFeTi high-entropy alloy with exceptional phase stability. Mater. Lett..

[11] Zhang Y (2013). High-entropy alloys with high saturation magnetization, electrical resistivity, and malleability. Sci. Rep..

[12] Nair RB (2018). Exceptionally high cavitation erosion and corrosion resistance of a high entropy alloy. Ultrason. Sonochem..

[13] Shuang S (2020). Corrosion resistant nanostructured eutectic high entropy alloy. Corros. Sci..

[14] Gorr B (2015). Phase equilibria, microstructure, and high temperature oxidation resistance of novel refractory high-entropy alloys. J. Alloy. Compd..

[15] Miracle DB, Senkov ON (2017). A critical review of high entropy alloys and related concepts. Acta Mater..

[16] Senkov ON (2010). Refractory high-entropy alloys. Intermetallics.

[17] Xia A, Franz R (2020). Thermal stability of MoNbTaVW high entropy alloy thin films. Coatings.

[18] Zhang C (2021). In situ study on the compression deformation of MoNbTaVW high-entropy alloy. J. Alloys Compds..

[19] Yang W (2021). Degradation properties of refractory MoNbTaVW high-entropy alloys with simultaneous Si/Al pack cementation coatings under high-temperature flame tests. Oxid. Met..

[20] Hudok D (1990). Properties and selection: Irons, steels, and high-performance alloys. Met. Handb..

[21] Sengupta A (1994). Tensile behavior of a new single-crystal nickel-based superalloy (CMSX-4) at room and elevated temperatures. J. Mater. Eng. Perform..

[22] Bo L (2021). Grain size and compositional gradient dependence of thermoelectric performance for Cu_3−x_Ni_x_SbSe_4_ materials. Results Phys..

[23] Sun SJ (2018). Ultrahigh cryogenic strength and exceptional ductility in ultrafine-grained CoCrFeMnNi high-entropy alloy with fully recrystallized structure. Mater. Today Nano.

[24] Rahman MH, Chowdhury EH, Hong S (2021). Nature of creep deformation in nanocrystalline cupronickel alloy: A molecular dynamics study. Results Mater..

[25] Hoang Giang N, Van Hoang V, Thi Thu Hanh T (2020). Melting of two-dimensional perfect crystalline and polycrystalline germanene. Phys. E Low Dimens. Syst. Nanostruct..

[26] Noori Z, Panjepour M, Ahmadian M (2015). Study of the effect of grain size on melting temperature of Al nanocrystals by molecular dynamics simulation. J. Mater. Res..

[27] Singh RN, Sommer F (1997). Segregation and immiscibility in liquid binary alloys. Rep. Prog. Phys..

[28] Huang X (2021). Atomistic simulation of chemical short-range order in HfNbTaZr high entropy alloy based on a newly-developed interatomic potential. Mater. Des..

[29] Jafary-Zadeh M (2017). Effect of chemical composition and affinity on the short- and medium-range order structures and mechanical properties of Zr–Ni–Al metallic glass. J. Non-Cryst. Solids.

[30] Tang C, Wong CH (2016). Formation of chemical short range order and its influences on the dynamic/mechanical heterogeneity in amorphous Zr–Cu–Ag alloys: A molecular dynamics study. Intermetallics.

[31] Senkov ON (2011). Mechanical properties of Nb_25_Mo_25_Ta_25_W_25_ and V_20_Nb_20_Mo_20_Ta_20_W_20_ refractory high entropy alloys. Intermetallics.

[32] Lee B-J (2001). Second nearest-neighbor modified embedded atom method potentials for bcc transition metals. Phys. Rev. B.

[33] Hammer B, Hansen LB, Nørskov JK (1999). Improved adsorption energetics within density-functional theory using revised Perdew–Burke–Ernzerhof functionals. Phys. Rev. B.

[34] Hirel P (2015). Atomsk: A tool for manipulating and converting atomic data files. Comput. Phys. Commun..

[35] Faken D, Jónsson H (1994). Systematic analysis of local atomic structure combined with 3D computer graphics. Comput. Mater. Sci..

[36] Wang S (2013). Atomic structure modeling of multi-principal-element alloys by the principle of maximum entropy. Entropy.

[37] Qi Y (1999). Molecular-dynamics simulations of glass formation and crystallization in binary liquid metals: Cu–Ag and Cu–Ni. Phys. Rev. B.

[38] Plimpton S (1995). Fast parallel algorithms for short-range molecular dynamics. J. Comput. Phys..

[39] Stukowski A (2009). Visualization and analysis of atomistic simulation data with OVITO—The Open Visualization Tool. Model. Simul. Mater. Sci. Eng..

